# Astrocytes require perineuronal nets to maintain synaptic homeostasis in mice

**DOI:** 10.1038/s41593-024-01714-3

**Published:** 2024-07-17

**Authors:** Bhanu P. Tewari, AnnaLin M. Woo, Courtney E. Prim, Lata Chaunsali, Dipan C. Patel, Ian F. Kimbrough, Kaliroi Engel, Jack L. Browning, Susan L. Campbell, Harald Sontheimer

**Affiliations:** 1 Department of Neuroscience, University of Virginia School of Medicine, Charlottesville, VA USA; 2 School of Neuroscience, Virginia Tech, Blacksburg, VA USA; 3 Department of Animal Sciences, Virginia Tech, Blacksburg, VA USA

**Keywords:** Neuroscience, Glial biology

## Abstract

Perineuronal nets (PNNs) are densely packed extracellular matrices that cover the cell body of fast-spiking inhibitory neurons. PNNs stabilize synapses inhibiting synaptic plasticity. Here we show that synaptic terminals of fast-spiking interneurons localize to holes in the PNNs in the adult mouse somatosensory cortex. Approximately 95% of holes in the PNNs contain synapses and astrocytic processes expressing Kir4.1, glutamate and GABA transporters. Hence, holes in the PNNs contain tripartite synapses. In the adult mouse brain, PNN degradation causes an expanded astrocytic coverage of the neuronal somata without altering the axon terminals. The loss of PNNs impairs astrocytic transmitter and potassium uptake, resulting in the spillage of glutamate into the extrasynaptic space. Our data show that PNNs and astrocytes cooperate to contain synaptically released signals in physiological conditions. Their combined action is altered in mouse models of Alzheimer’s disease and epilepsy where PNNs are disrupted.

## Abstract (web summary)

Perineuronal nets stabilize synapses inhibiting synaptic plasticity. Here, the authors show that perineuronal nets act as a diffusion barrier facilitating astrocytic clearance of synaptically released ions and neurotransmitters.

## Main

Excitatory synapses predominantly associate with astrocytic processes^1^, allowing astrocytes to sense and modulate synaptic activity^2^. Such tripartite synapses, place astrocytic processes perfectly to remove neurotransmitters and ions released in conjunction with synaptic activity, particularly ensuring that glutamate (Glu) does not spill out of synapses and activate extrasynaptic receptors^3^ causing excitotoxicity^4^.

Neurons and astrocytes are embedded in the extracellular matrix (ECM) composed of proteoglycans, glycoproteins and polysaccharides, including hyaluronan, chondroitin sulfate proteoglycans aggrecan, brevican, versican and neurocan, tenascin and link proteins such as CRTL1 and BRAL2 (refs. ^5,6^). Some ECM constituents can interact with synaptic receptors and ion channels thereby affecting synaptic vesicle release, dendritic spine morphology and structural integrity of synapses^7,8^. Moreover, the ECM alters diffusion of ions in the extracellular space^9,10^.

A highly condensed form of ECM forms a corset-like structure known as PNNs mostly around parvalbumin (PV) expressing inhibitory neurons. Easily recognized by the binding of wisteria floribunda agglutinin (WFA)^7^, PNNs encapsulate the cell soma, dendrites and axon initial segment. PNNs stabilize synapses and restrict synaptic plasticity, particularly in pathways exhibiting developmental activity-dependent plasticity such as the visual system^11^; however, whether PNNs interact structurally and functionally with astrocytes at tripartite synapses has not been studied.

Here we show synapses onto PNN-expressing fast-spiking neurons (FSNs) occupying small perforations or holes within the PNNs, where excitatory and inhibitory synapses co-mingle with astrocytic processes, hence forming tripartite synapses. PNN disruption impairs astrocytic uptake of synaptically released Glu and K^+^ and causes spillage of Glu into the perisynaptic space. PNN disruption is also associated with expansion of astrocytic leaflets across the cell body, yet synapses retain their place. These observations suggest a previously unreported function of PNNs, namely, to create a barrier that limits diffusion of Glu and K^+^ from the synaptic cleft so as to synergize with astrocytes for effective reuptake of neuronally released ions and transmitters. Hence, PNNs are important structural and functional contributors to the tripartite synapse.

## Results

### Astrocytic processes express homeostatic proteins in PNN holes

Synaptic spines on excitatory neurons are ensheathed by astrocytic processes called leaflets^12^, which facilitate the clearance of synaptically released ions and neurotransmitters. On inhibitory neurons, synapses form mainly on the somata where PNNs form a dense coat. Small holes in the PNN provide the only access for synaptic terminals. Therefore, our first question was whether such axosomatic synapses on inhibitory neurons are tripartite synapses and contain astrocytic processes associated with the synapse.

**Fig. 1:**
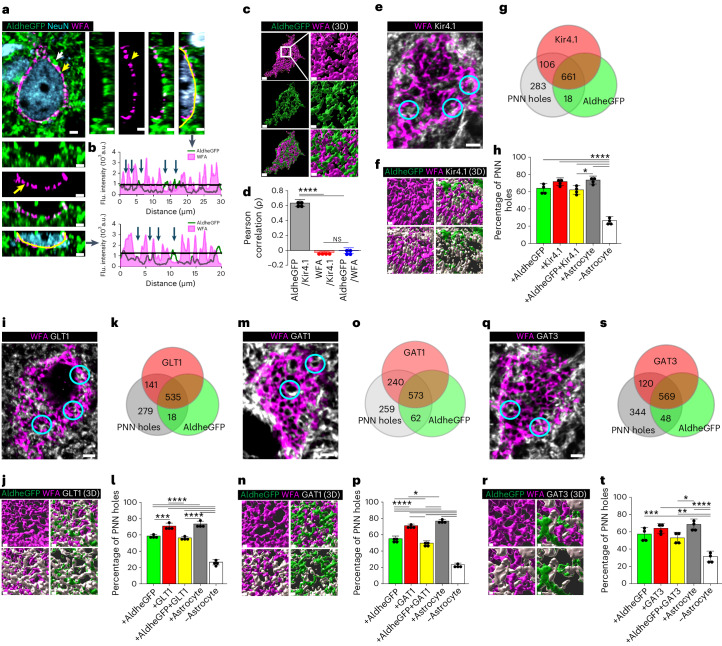
Astrocytic processes express homeostatic proteins in PNN holes in adult mice. **a**,**b**, Confocal micrograph (**a**) showing PNN (WFA) and astrocytic processes (AldheGFP). White and yellow arrows show astrocytic processes on and within PNN holes, respectively. Side and bottom panels show orthogonal planes and line intensity profiles (**b**) of WFA and AldheGFP showing astrocytic processes in PNN holes (blue arrows). Black line shows the WFA threshold. **c**, 3D reconstruction of PNN (WFA) and astrocytic processes (AldheGFP) showing a non-overlapping interdigitating interface on the neuronal surface. Scale bars, 2 µm (left), 1 µm (right). **d**, Positive Pearson correlation between astrocytic markers (AldheGFP and Kir4.1) that show lack of correlation with PNN (WFA). *n* = 4 mice per group. **e**,**f**, IHC (**e**) and 3D reconstruction (**f**) of astrocytic processes (AldheGFP) expressing Kir4.1 in PNN (WFA) holes. Representative holes are encircled. **g**,**h**, Proportional occupancy (**g**), and percentage of PNN holes (**h**) containing AldheGFP, Kir4.1, both, any astrocytic marker and empty. *n* = 40 PNNs/4 mice per group. **i**,**j**, Confocal micrograph (**i**) and 3D reconstruction (**j**) of astrocytic processes (AldheGFP) expressing GLT1 in PNN (WFA) holes. Representative holes are encircled. **k**,**l**, Proportional occupancy (**k**) and percentage of PNN holes (**l**) containing AldheGFP, GLT1, both, occupied by any astrocytic marker and empty. *n* = 40 PNNs/4 mice per group. **m**,**n**, IHC (**m**) and 3D reconstruction (**n**) of astrocytic processes (AldheGFP) expressing GAT1 in PNN (WFA) holes. Representative holes are encircled. **o**,**p**, Proportional occupancy (**o**) and percentage of PNN holes (**p**) containing astrocytic processes expressing AldheGFP, GAT1, both, any astrocytic marker and empty. *n* = 40 PNNs/4 mice per group. **q**,**r**, IHC (**q**) and 3D reconstructions (**r**) of astrocytic processes (AldheGFP) expressing GAT3 in PNN (WFA) holes. Representative holes are encircled. **s**,**t**, Proportional occupancy (**s**) and percentage of PNN holes (**t**) containing astrocytic processes (AldheGFP), GAT3, both, any astrocytic marker and empty. *n* = 40 PNNs/4 mice per group. Bar data show mean ± s.d. Dots represent data points. ****P < 0.0001; ***P < 0.001, **P < 0.01, *P < 0.05, NS, not significant (*P* > 0.05). One-way analysis of variance (ANOVA) and Tukey’s post hoc test (**d**,**h**,**l**,**p**,**t**). Scale bars, 2 µm (**a**,**e**,**i**,**m**,**q**) and 1 µm (**c**,**f**,**j**,**n**,**r**). Both male and female mice were used. Flu., fluorescence. a.u., arbitrary unit. Source data

**Extended Data Fig. 1:**
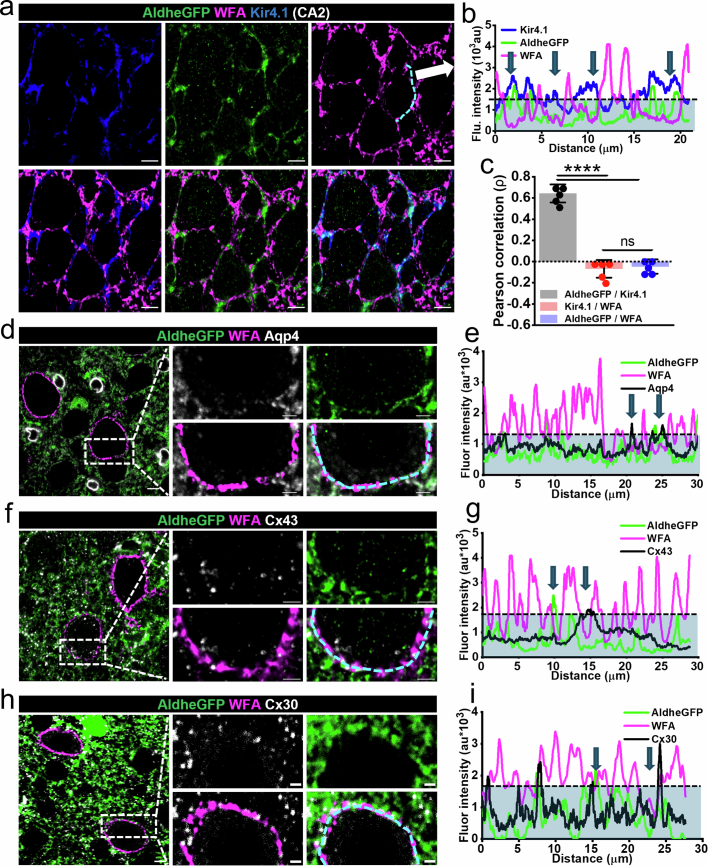
PNNs holes contain astrocytic processes in hippocampal CA2 and low-expressing astrocytic homeostatic proteins in PNN holes in cortex. **a**, IHC showing hippocampal CA2 PNNs (WFA) and astrocytic processes (AldheGFP, Kir4.1). Scale 5 µm. **b** Intensity profiles of a line (Fig. a (arrow)), showing fluorescence intensity of PNN and astrocytic markers. Blue arrows point to PNN holes occupied by astrocytic processes expressing Kir4.1. **c**, Pearson correlation between astrocytic markers AldheGFP and Kir4.1 and negative correlation between PNN marker (WFA) and astrocytic markers Kir4.1 and AldheGFP. N = 5 mice per group. ****P < 0.0001, ns - not significant, P > 0.05. One-way ANOVA with Tukey’s post-hoc test. **d**, IHC showing aquaporin 4 expression (Aqp4) in astrocytic processes (AldheGFP) in cortical PNN holes (WFA). **e**, Intensity profiles of the line drawn on PNN (bottom right d) representing PNN holes (blue arrows) occupied with astrocytic processes (AldheGFP) expressing Aqp4. **f**, IHC showing connexin 43 expression (Cx43) in astrocytic processes (AldheGFP) in cortical PNN holes (WFA). **g**, Intensity profiles of line drawn on PNN (bottom right f) representing PNN holes (blue arrows) occupied with astrocytic processes (AldheGFP) expressing connexin 43. **h**, IHC showing connexin 30 expression (Cx30) in astrocytic processes (AldheGFP) in cortical PNN holes (WFA). **i**, Intensity profiles of line drawn on the PNN (bottom right h) representing PNN holes (blue arrows) occupied with astrocytic processes (AldheGFP) expressing connexin 30. The dotted black line and blue area under it in b, e, g, and i, represent WFA threshold to delineate PNN holes. Scale 5 µm in large images, 2 µm in magnified images in d, f, and h. Source data

We visualized astrocytic leaflets in PNN holes, using adult male and female FVB-N//Swiss Webster-Aldh1l1-eGFP mice in relationship to WFA-labeled PNNs in layers 3–4 (L3–4) of the somatosensory cortex (SSC). This area has the highest PNN density^13^, with a vast majority of astrocytes contacting PNNs. Confocal images show a majority of astrocytic leaflets terminating within PNN holes with only a few terminating on distal side of the PNN (white and yellow arrows Fig. 1a). We rarely observed astrocytic processes interspaced between PNN and cell body. Intensity profiles along the PNNs (Fig. 1a and Extended Data Fig. 1) show peaks of astrocytic AldheGFP (Fig. 1b, green line) in the holes of PNNs (with lowest WFA signal, magenta area) suggesting astrocytes preferentially occupy PNN holes (Fig. 1b). The three-dimensional (3D) rendering (Fig. 1c) and the Pearson correlation analysis of the PNN marker WFA with astrocytic AldheGFP and Kir4.1 (Fig. 1d) show no correlation between WFA and both astrocytic markers, consistent with a non-overlapping interdigitating spatial interface where astrocytic processes are almost exclusively found in the PNN holes.

While PNNs typically associate with inhibitory neurons, PNNs also surround excitatory CA2 pyramidal neurons. Repeating the above analysis on hippocampus CA2 shows PNN holes similarly occupied by astrocytic processes (Extended Data Fig. 1a,b) where WFA and astrocytic AldheGFP or Kir4.1 show a similar lack of correlation by the Pearson analysis (Extended Data Fig. 1c).

Because astrocytic processes are mainly confined to PNN holes, astrocytic proteins serving potassium, glutamate and GABA uptake at synapses may also localize to PNN holes. Using immunohistochemistry (IHC) analyzed by high-magnification confocal imaging and line-intensity profile analysis^14,15^ (Supplementary Fig. 1), we quantified expression of Kir4.1, GLT1 and GABA transporters GAT1 and GAT3 along with astrocytic AldheGFP in >900 holes in each experimental group. AldheGFP, Kir4.1 and both were found in 63%, 71% and 62% of PNN holes, respectively (Fig. 1e–h). In a separate set of experiments, we observed 59%, 70% and 56% PNN holes with AldheGFP, GLT1 and both immunoreactivities, respectively (Fig. 1i–l). In similar proportions, astrocytic processes in PNN holes expressed both GABA transporters, GAT1 (Fig. 1m–p) and GAT3 (Fig. 1q–t); however, Aqp4, Cnx43 and Cnx30, abundant astrocyte proteins were almost undetectable in PNN holes (Extended Data Fig. 1d–i). These data suggest that astrocytic processes in PNN holes selectively express proteins that support the clearance of synaptically released ions and transmitters.

### PNN holes house tripartite synapses

We next asked whether astrocytic processes coexist with synapses in the PNN holes and whether holes exclusively house excitatory or inhibitory synapses and if so, whether astrocytes show matching Glu or GABA transporters, respectively.

**Fig. 2:**
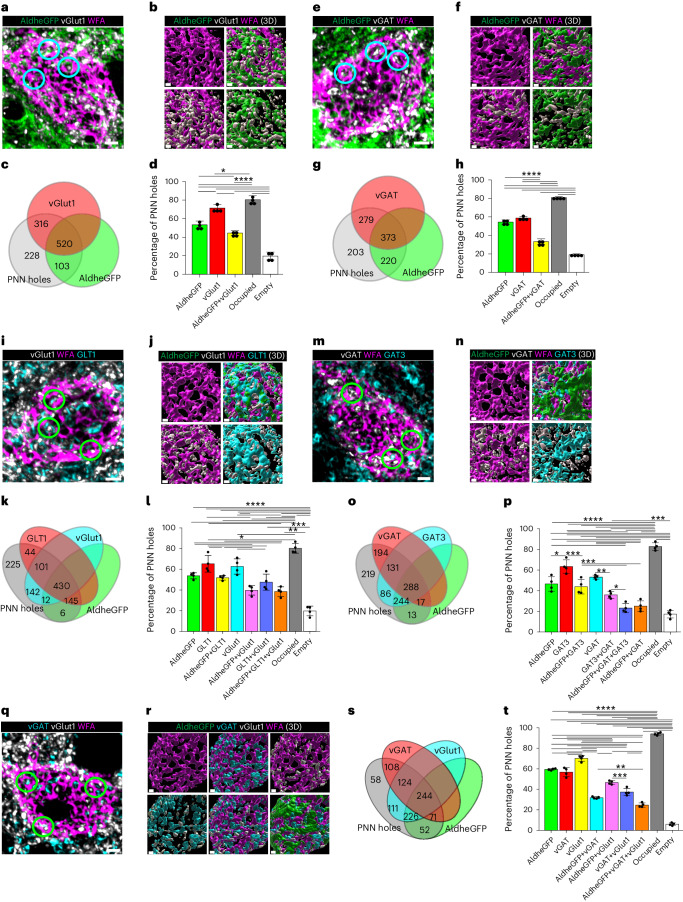
Cortical PNN holes contain synapses and astrocytic processes that express neurotransmitter transporters in adult mice. **a**,**b**, IHC (**a**) and 3D reconstruction (**b**) of astrocytic processes (AldheGFP) and excitatory presynaptic terminals (vGlut1). Representative holes containing both are encircled. **c**,**d**, Proportional occupancy (**c**), and percentage of PNN holes (**d**) containing astrocytic processes (AldheGFP), excitatory synapses (vGlut1), both and none. *n* = 40 PNNs/4 mice per group. **e**,**f**, IHC (**e**) and 3D reconstruction (**f**) of astrocytic processes (AldheGFP) and inhibitory presynaptic terminals (vGAT) in PNN holes. Representative holes containing both are encircled. **g**,**h**, Proportional occupancy (**g**) and percentage of PNN holes (**h**) containing astrocytic processes (AldheGFP), inhibitory synapses (vGAT), both and none. *n* = 40 PNNs/4 mice per group. **i**,**j**, IHC (**i**) and 3D reconstruction (**j**) of astrocytic processes (AldheGFP) expressing glutamate transporter (GLT1), excitatory presynaptic terminals (vGlut1), both and none in PNN holes. Representative holes containing all are encircled. **k**,**l**, Proportional occupancy (**k**), and percentage of PNN holes (**l**) containing astrocytic processes (AldheGFP), glutamate transporters (GLT1), excitatory synapses (vGlut1) and various combinations in PNN holes. *n* = 40 PNNs/4 mice per group. **m**,**n**, IHC (**m**), and 3D reconstruction (**n**) of PNN (WFA) holes containing astrocytic processes (AldheGFP) expressing GABA transporter (GAT3), and inhibitory synapses (vGAT). Representative holes containing all are encircled. **o**,**p**, Proportional occupancy (**o**), and percentage of PNN holes (**p**) containing astrocytic processes (AldheGFP) expressing GABA transporter (GAT3), inhibitory synapses (vGAT), and various combinations. *n* = 40 PNNs/4 mice per group. **q**,**r**, IHC (**q**), and 3D reconstruction (**r**) of PNN (WFA) holes containing inhibitory (vGAT) and excitatory (vGlut1) synapses and astrocytic processes (AldheGFP). Representative PNN holes containing vGlut1 and vGAT are encircled. **s**,**t**, Proportional occupancy (**s**) and percentage of PNN holes (**t**) containing astrocytic processes (AldheGFP), inhibitory (vGAT) and excitatory (vGlut1) synapses in various combinations. *n* = 40 PNNs/4 mice per group. Bar data represent mean ± s.d. ****P < 0.0001; ***P < 0.001, **P < 0.01, *P < 0.05. One-way ANOVA, Tukey’s post hoc test (**d**,**h**,**l**,**p**,**t**). Scale bars, 2 µm (**a**,**e**,**i**,**m**,**q**) and 1 µm (**b**,**f**,**j**,**n**,**r**). Both male and female mice were used. Source data

**Extended Data Fig. 2:**
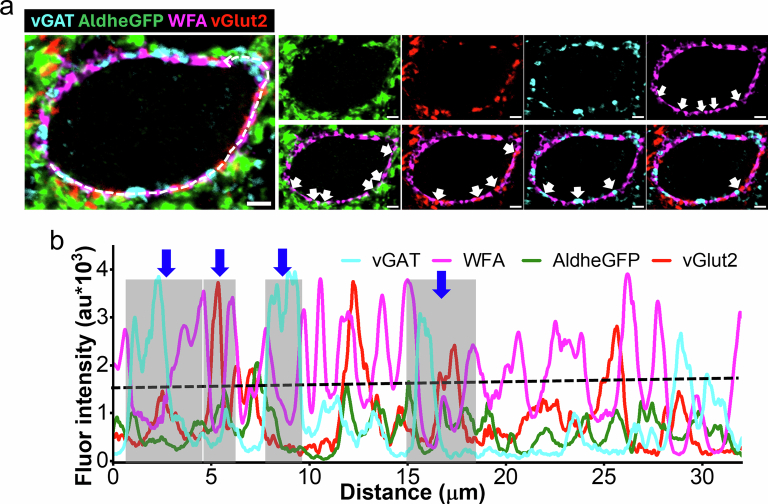
PNN holes contain astrocytic processes and vGlut2-expressing thalamocortical synapses. **a**, Representative confocal micrographs showing vGlut2-expressing synapses, astrocytic processes (AldheGFP) and inhibitory synapses (vGAT) in cortical PNN holes (WFA). White arrows point to the PNN holes occupied by specific markers. Scale 2 µm. **b**, Line intensity profiles of various markers generated by drawing a polyline on the PNN (white dotted line drawn in large image in a). The dotted black line represents the WFA threshold. A few represented PNN holes are marked by blue arrows and the area of the holes is marked by gray bars. Line graphs of vGlut2, AldheGFP and vGAT showing a peak within the gray bar area suggest their presence in the PNN holes. Source data

We analyzed >1,000 PNN holes regarding distribution of excitatory and inhibitory synaptic terminals in conjunction with the astrocytic marker AldheGFP on PNN-expressing cortical FSNs in adult male and female FVB-N//Swiss Webster-Aldh1l1-eGFP mice (Fig. 2). Approximately 80% of PNN holes contained astrocytic leaflets, vGlut1 terminals or both, leaving only around 20% holes unoccupied (Fig. 2a–d). A total of 71% of all PNN holes contained excitatory synapses (vGlut1), 53% contained astrocytic processes (AldheGFP) and 44% showed co-occupancy of excitatory synapses with astrocytic processes (Fig. 2c,d and Supplementary Video 1). Hence, over 60% of synaptic contacts contained astrocytic processes. PNN holes also contained vGlut2-expressing synaptic terminals from thalamocortical sensory projections, along with astrocytic leaflets (Extended Data Fig. 2a,b).

Inhibitory synaptic terminals in PNN holes showed a lower astrocytic occupancy (58%), with only 33% of all PNN holes showing both astrocytic and synaptic components (Fig. 2e–h); however, similar to the vGlut1 terminals, 58% of vGAT-occupied holes showed astrocytic contacts, and overall 80% of PNN holes were occupied by either astrocytic processes, vGAT terminals or both (Fig. 2g,h and Supplementary Video 2).

To determine whether astrocytic leaflets colocalizing with synapses in PNN holes also express Glu and GABA transporters, we analyzed >1,000 PNN holes. Approximately 47% contained vGlut1 terminals and GLT1-expressing astrocytic leaflets (Fig. 2i–l), suggesting that 75% of all excitatory terminals are accompanied by astrocytic processes equipped to uptake synaptically released Glu. Approximately 35% of holes contained both vGAT terminals and GAT3-expressing astrocytic leaflets, suggesting that 67% of inhibitory terminals in PNN holes have astrocytes capable of GABA uptake (Fig. 2m–p). Approximately 80% of holes were occupied by one or more elements from astrocytes or synapses or both (Fig. 2o,p), and only 20% of holes displayed no label.

PNN holes may contain excitatory or inhibitory synapses or both. IHC data suggests that vGlut1 and vGAT terminals were coexpressed in 37% of all holes and 64% contained astrocytic processes (Fig. 2q–t). Only 33% and 19% of holes contained either vGlut1 or vGAT terminals. vGAT and vGlut2 also colocalized in PNN holes of glutamatergic synapses from thalamocortical sensory projections (Extended Data Fig. 2a,b). Notably, combining markers of glutamatergic and GABAergic synapses with astrocytes increased the overall occupancy of PNN holes from around 80% (Fig. 2d,h,l,p) to 95% (Fig. 2t) suggesting that nearly all PNN holes are occupied with a mixture of synapses and astrocytic processes.

Taken together, PNN holes contain both excitatory and inhibitory synapses the majority of which are accompanied by astrocytic leaflets expressing both Glu and GABA transporters, suggesting that PNN holes house a structural and functional analog of tripartite synapses. Each PNN hole may contain excitatory, inhibitory or both types of synaptic terminals. Finally, almost all PNN holes are filled with a synapse and astrocytic processes, rarely leaving holes unoccupied.

### PNN restricts astrocytic coverage and synapses on PV neurons

**Fig. 3:**
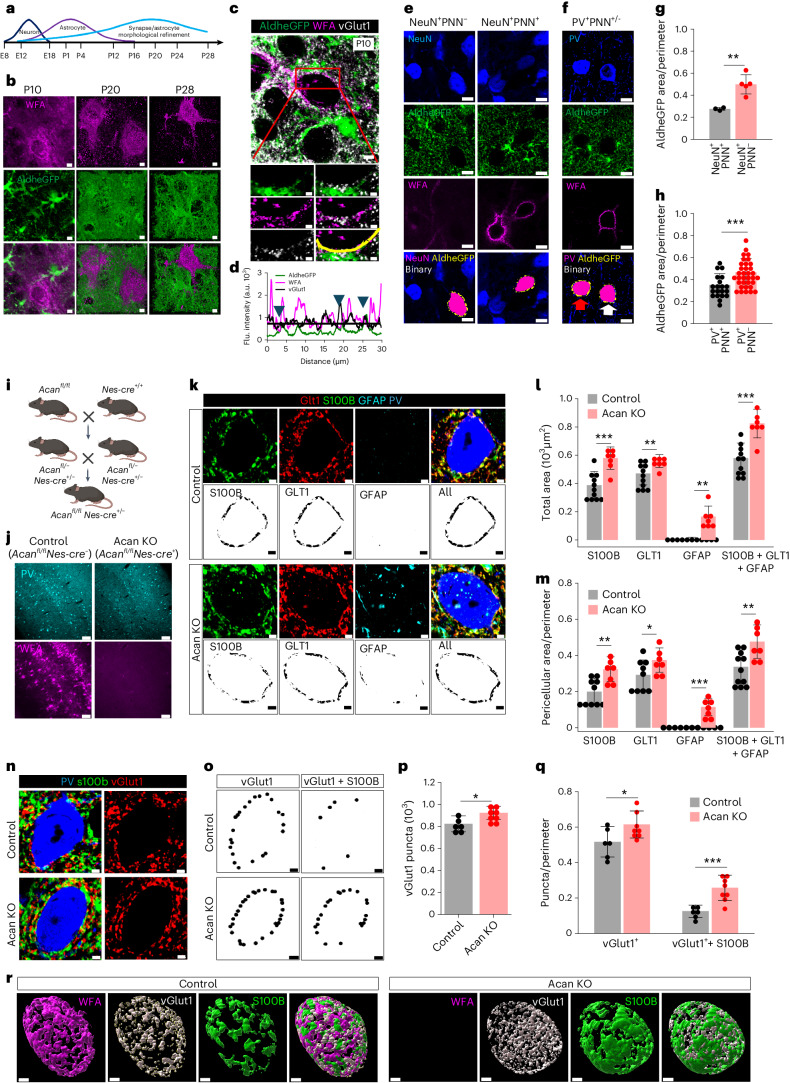
Developmental PNN maturation restricts astrocytic coverage and tripartite synapses. **a**, Developmental timeline in mouse brain. **b**, IHC of cortical PNN (WFA) and astrocytes (AldheGFP) on postnatal days 10, 20 and 28 showing concurrent PNN and astrocyte maturation. Scale bars, 2 µm. **c**, IHC of cortical PNN (WFA), astrocytic processes (AldheGFP) and synapses (vGlut1) in P10 mice. Scale bars, 5 µm (top) and 2 µm (bottom). **d**, Line intensity profile of PNN (line in **c**) showing vGlut1 and AldheGFP peaks in PNN holes (arrows). Black line represents WFA threshold. **e**,**f**, IHC and binary (bottom) images showing cortical neurons (**e**) and PV neurons (**f**) with or without PNN (WFA) and their astrocytic coverage (AldheGFP). White and red arrows in **f** point to PNN-expressing and non-expressing PV neurons, respectively. Scale bars, 5 µm. **g**, Lower astrocytic (AldheGFP) coverage in cortical NeuN^+^PNN^+^ neurons (*n* = 23 cells/3 mice) than NeuN^+^PNN^−^ neurons (*n* = 27 cells/5 mice). **P = 0.0038. **h**, Lower astrocytic (AldheGFP) coverage of cortical PV^+^PNN^+^ neurons (*n* = 20 cells/4 mice) than PV^+^PNN^−^ neurons (*n* = 34 cells/7 mice). ***P = 0.0006. **i**,**j**, Generation of Acan-knockout (KO; *Acan*^*f*^^*l/f*^^*l*^*Nes-cre*^+^) mice (**i**) and validation of PNN knockout by IHC of PNN (WFA) and PV (**j**). Scale bars, 100 µm. Illustration in **i** created using BioRender. **k**, IHC (top) and binary (bottom) images of astrocytic coverage with S100B, GLT1 and GFAP on cortical PV neurons in control and Acan-knockout mice. Scale bars, 2 µm. **l**, Higher total astrocytic area in Acan-knockout mice with astrocytic markers S100B (***P = 0.0003), GLT1 (**P = 0.0097), GFAP (**P = 0.0013) and combined (S100B + GLT1 + GFAP) (***P = 0.0002) (*n* = 11 mice (control), 7 mice (KO)). **m**, Higher pericellular astrocytic coverage of PV neurons in Acan-knockout mice with astrocytic markers S100B (**P = 0.0029), GLT1 (*P = 0.039, *n* = 10 m (control), 7 m (KO); GFAP (***P = 0.0007) and combined (S100B + GLT1 + GFAP) of all (**P = 0.0082) (*n* = 11 mice (control), 7 mice (KO)) in S100B, GFAP and combined groups. **n**, IHC of cortical PV neuron, glutamatergic synapses (vGlut1) and astrocytes (S100B) in Acan-knockout mice. Scale bars, 2 µm. **o**, Binary images showing total pericellular synaptic puncta (vGlut1) and puncta with astrocytic processes (vGlut1 + S100B) in control and Acan-knockout mice. **p**, Higher total vGlut1 puncta in Acan-knockout cortex (*P = 0.0207). *n* = 6 mice (control), 8 mice (KO). **q**, Altered pericellular vGlut1 puncta (*P = 0.0497) and puncta with S100B processes (vGlut1 + S100B) (***P = 0.0008) on PV neurons in Acan-knockout mice (*n* = 6 mice (control), 8 mice (KO). **r**, 3D reconstruction of PNN (WFA), pericellular astrocytic coverage (S100B) and excitatory synapses (vGlut1) in control and Acan-knockout cortex. Scale bars, 2 µm. Bar data represent mean ± s.d. Dots represent data points. Unpaired two-tailed *t*-test (**g**,**h**,**l**,**m**,**p**,**q**). We used adult male and female mice, unless age is specified, as in **b**–**d**. Source data

PNN deposition in the developing brain coincides with the closure of critical periods of plasticity believed to exclude synapses from future modifications^7,11^. In the developing brain, the morphological maturation of astrocytes^16^ occurs in tandem with the condensation of PNNs^17^ (Fig. 3a,b) during which astrocytic processes and synaptic contacts can both be traced to the newly formed PNN holes (Fig. 3c,d). Based on the concurrent development (Fig. 3b) and our findings that PNN holes contain both synapses and astrocytic processes (Figs. 1 and 2), we hypothesized that PNNs also stabilize astrocytic leaflets at PNN holes thereby limiting the astrocytic coverage on PNN-expressing neurons to only those patches of membrane accessible through the PNN perforations.

To test this hypothesis, we used adult male and female FVB-N//Swiss Webster-Aldh1l1-eGFP mice and compared pericellular astrocytic coverage of PNN-expressing and non-expressing neurons identified by NeuN, AldheGFP and WFA (Fig. 3e and Supplementary Fig. 2). Within a 0.8-µm perimeter of the cell soma, PNN-expressing (NeuN^+^WFA^+^) neurons showed a significantly lower astrocytic coverage than PNN-lacking (NeuN^+^WFA^−^) neurons (Fig. 3e,g). To confirm that the lower pericellular astrocytic coverage around PV neurons is attributed to the PNN, we compared the astrocytic coverage of PNN-expressing PV neurons (PV^+^PNN^+^) with a rare cortical population of PNN-lacking PV neurons (PNN^−^PV^+^). PNN-lacking PV neurons (PV^+^PNN^−^) had significantly higher astrocytic coverage than PNN-expressing PV neurons (PV^+^PNN^−^) (Fig. 3f,h), confirming that pericellular astrocytic coverage negatively correlates with the presence of the PNN, as PNNs restricts access of astrocytes to holes within the PNN.

**Extended Data Fig. 3:**
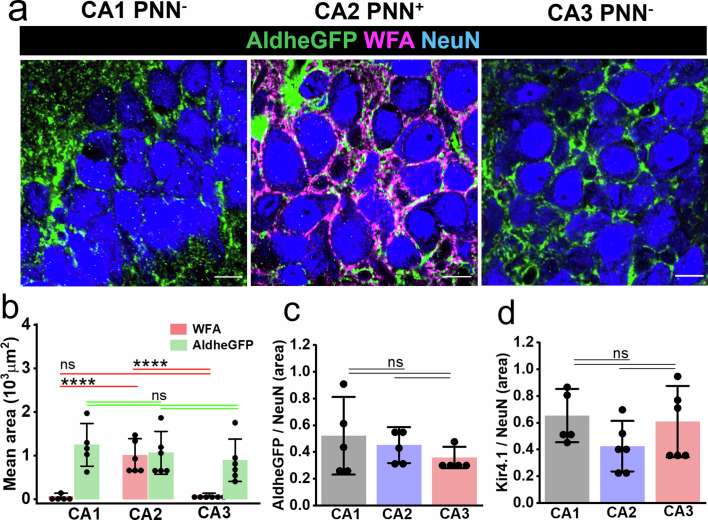
Astrocytic coverage on PNN-expressing CA2 neuron is not different from PNN-lacking CA1 and CA3 neurons. **a**, IHC showing NeuN, AldheGFP, and WFA fluorescence in hippocampal CA1, CA2, and CA3 stratum pyramidale. Scale bar 10 µm. **b**, Bar graph representing the mean total coverage area of AldheGFP and WFA in CA1, CA2, and CA3 areas. Due to PNNs, WFA covered area in CA2 is significantly higher compared to CA1 (****P < 0.0001) and CA3 (****P < 0.0001); however, AldheGFP covered area in CA2 is statistically indifferent from CA1 (P = 0.8154) and CA3 (P = 0.8160). n = 5 mice in CA1, 6 mice in CA2 and CA3 groups. Red and green lines show comparisons between red (WFA) bars and green bars (AldheGFP) respectively. ns – P > 0.05. **c**, Bar graph showing non-significant total astrocytic area (AldheGFP) normalized to neuronal area (NeuN) in CA1, CA2, and CA3 regions (CA1 vs. CA2 (P = 0.8318), CA1 vs. CA3 (P = 0.3929), CA2 vs. CA3 (P = 0.7253)). n = 5 mice per group. ns – P > 0.05. **d**, Bar graph showing non-significant total astrocytic area (Kir4.1) normalized to neuronal area (NeuN) in CA1, CA2, and CA3 regions (CA1 vs. CA2 (P = 0.2403), CA1 vs. CA3 (P = 0.9423), CA2 vs. CA3 (P = 0.5301)). n = 5(CA1), and 6 (CA2, CA3) mice, ns – P > 0.05. Bar data represents ± SD; dots represent data points. One-way ANOVA, Tukey’s post-hoc test in b–d. Source data

As mentioned above, excitatory neurons in CA2 express a less-condensed form of PNNs^18^. Astrocytic coverage of these CA2 neurons, visualized by using astrocytic markers AldheGFP and Kir4.1, did not differ from their CA1 and CA3 counterparts lacking PNNs (Extended Data Fig. 3a–d) suggesting that only the typical condensed PNNs on cortical PV neurons leads to restricted pericellular astrocytic coverage.

**Extended Data Fig. 4:**
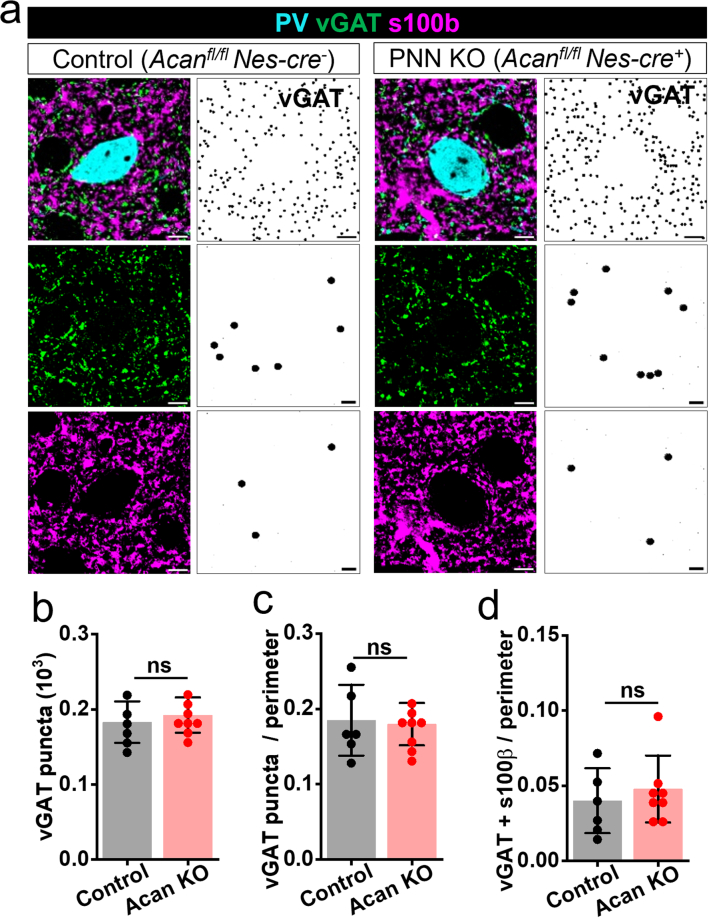
Inhibitory synapses remain unchanged in Acan KO mice. **a**, IHC (left panels) showing PV, vGAT and s100b immunofluorescence in control (*Acan*^*fl/fl*^ *Nes-cre*^*-*^) and Acan KO (*Acan*^*fl/fl*^ *Nes-cre*^*+*^) mouse cortex. The right panels show total binary puncta of vGAT (top), pericellular vGAT puncta (middle) and pericellular vGAT puncta with astrocytic contacts (bottom) around PV neurons. Scale bar 5 µm. **b–d**, Bar diagrams showing unaltered (**b**) total vGAT puncta (P = 0.5155), (**c**) pericellular density of vGAT puncta (P = 0.8164), and (**d**) pericellular density of vGAT puncta with astrocytic contacts (P = 0.5220) in acan KO mice with no PNNs. n = 6 mice (control), 8 mice (Acan KO)) in **b**–**d**, Unpaired two-tailed t-test in **b**–**d**. Bar data represent mean ± SD; dots represent data points. Source data

If PNN deposition at the end of the critical period restricts the pericellular coverage by synapses and astrocytic processes, then a lack of PNN during development should allow astrocytic leaflets and synaptic contacts to occupy larger surface areas on PV neurons. To address this question, we generated Acan-knockout (*Acan*^*fl/fl*^ *Nes-cre*^*+*^) mice (Fig. 3i) that never deposit PNNs (Fig. 3j). Using multiple markers of astrocytes (S100B, GLT1 and GFAP), we observed a significantly higher total (Fig. 3k,l) and pericellular (Fig. 3k,m) coverage of astrocytic leaflets around cortical PV neurons in adult Acan-knockout mice of both sexes. Interestingly, lack of PNN deposition increased the overall (Fig. 3n,p) and pericellular vGlut1 expressing synaptic contacts (Fig. 3o,q,r). The increased synaptic contacts seem to retain their tripartite nature as suggested by a significantly higher number of vGlut1 synapses contacting astrocytic processes (Fig. 3o,q,r). The vGAT-expressing synaptic contacts did not show any alterations (Extended Data Fig. 4a,d). These data suggest that PNN deposition during development limits the pericellular density of synapses and area of astrocytic occupancy consistent with PNNs playing a pivotal role in the developmental maturation of astrocytes as well as perisomatic tripartite synapses.

### PNN disruption increases astrocytic coverage of neuronal somata

**Fig. 4:**
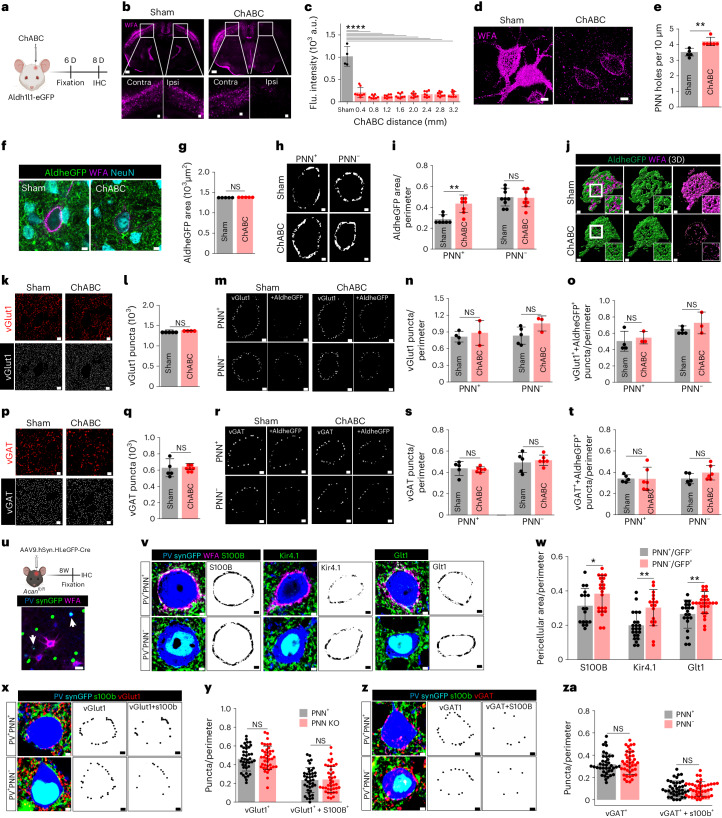
PNN disruption increases pericellular astrocytic coverage without altering tripartite synapses in adult mice. **a**, Experimental outline. **b**, IHC showing cortical PNN disruption on ChABC injection. Scale bars, 1 mm top, 100 µm bottom. **c**, Reduced WFA intensity in ChABC-injected mice cortex (****P < 0.0001, sham versus ChABC), *n* = 4 mice (sham), 8 mice (ChABC). **d**, IHC showing disrupted cortical PNN (WFA) on ChABC injection. Scale bars, 5 µm. **e**, Increased PNN holes on cortical PNN degradation (**P = 0.0019, *n* = 6 mice per group). **f**, IHC showing AldheGFP, NeuN and WFA fluorescence in cortex from sham and ChABC-injected mice. Scale bars, 5 µm. **g**, Unchanged total cortical AldheGFP area on ChABC injection (*P* = 0.9574, *n* = 5 mice per group). **h**, Binary images showing pericellular astrocytic coverage of AldheGFP in cortex. **i**, Increased pericellular AldheGFP coverage of cortical PNN^+^ neurons on ChABC injection (*P = 0.0025, *n* = 7 mice per group), however, remained unaltered in PNN^−^ neurons (*P* = 0.8510, *n* = 9 mice (control), 8 mice (ChABC). **j**, 3D reconstruction showing increased astrocytic coverage on PNN digestion. **k**, IHC (top) and binary puncta (bottom) of vGlut1 synapses in sham and ChABC-injected mice cortex. Scale bars, 5 µm. **l**, Unaltered vGlut1 puncta in ChABC-injected cortex (*P* = 0.6357, *n* = 4 mice (sham), 5 mice (ChABC). **m**, Binary puncta of pericellular vGlut1 and vGlut1 with astrocyte (+AldheGFP) in PNN^+^ and PNN^−^ cortical neurons in sham and ChABC-treated mice. **n**, Unaltered pericellular vGlut1 puncta in ChABC-treated mice around PNN^+^ (*P* = 0.6546, *n* = 4 mice (sham), 3 mice (ChABC)) and PNN^−^ (*P* = 0.0930, *n* = 5 mice (sham), 3 mice (ChABC)) neurons. **o**, Unaltered pericellular synaptic puncta with astrocytic contact (vGlut1 + AldheGFP) in ChABC-treated mice around PNN^+^ (*P* = 0.6242, *n* = 4 mice (sham), 3 mice (ChABC)) and PNN^−^ (*P* = 0.4345, *n* = 5 mice (sham), 3 mice (ChABC)) neurons. **p**, IHC (top) and binary puncta (bottom) of vGAT fluorescence in sham and ChABC-injected mice cortex. Scale bars, 5 µm. **q**, Unaltered vGAT puncta in ChABC-injected mice (*P* = 0.7763, *n* = 5 mice (sham), 7 mice (ChABC)_. **r**, Binary puncta of pericellular vGAT and vGAT with astrocyte (+AldheGFP) around PNN^+^ and PNN^−^ cortical neurons in sham and ChABC-treated mice. **s**, Unaltered pericellular vGAT puncta in ChABC-treated mice around PNN^+^ (*P* = 0.7378) and PNN^−^ neurons (*P* = 0.6734). *n* = 5 mice (sham), 6 mice (ChABC)) in both. **t**, Unaltered pericellular synaptic puncta with astrocytic contact (vGAT^+^ + AldheGFP^+^) in ChABC-treated mice around PNN^+^ (*P* = 0.9640) and PNN^−^ (*P* = 0.1464) neurons. *n* = 5 mice (sham), 6 mice (ChABC) in both. **u**, AAV9-mediated Acan KO and IHC confirmation of PNN deletion on PV neurons (arrows). Scale bars, 20 µm. **v**, IHC (left) and binary (right) images showing astrocytic coverage (S100B, Kir4.1, GLT1) of PV^+^PNN^+^ and PV^+^PNN^−^ neurons in SynCreGFP-injected *Acan*^*fl/fl*^ mice. **w**, Increased pericellular coverage of astrocytic markers S100B (*P = 0.0442, *n* = 17 cells/3 mice (PNN^+^), 22 cells/3 mice (PNN^−^), Kir4.1 (**P = 0.0047, *n* = 23 cells/3 mice (PNN^+^), 17 cells/3 mice (PNN^−^) and GLT1 (**P = 0.0021, *n* = 20 cells/3 mice (PNN^+^), 26 cells/3 mice (PNN^−^), on PV neurons with PNN knockout. **x**, IHC (left) and binary images of pericellular vGlut1 puncta (middle) and vGlut1 puncta with astrocytic processes (vGlut1 + S100B) (right) in PV^+^PNN^+^ and PV^+^PNN^−^ cortical PV neurons. **y**, Unaltered pericellular vGlut1 puncta (*P* = 0.6902) and pericellular vGlut1 puncta with astrocytic processes (vGlut1 + S100B) (*P* = 0.8284) on PV neurons with PNN knockout using AAVSynCreGFP (*n* = 40 cells/4 mice per group). **z**, IHC (left) and binary images of pericellular vGAT puncta (middle), and vGlut1 puncta with astrocytic processes (vGAT + S100B) (right) in PV^+^PNN^+^ and PV^+^PNN^−^ cortical PV neurons. **za**, Unaltered pericellular vGAT puncta (*P* = 0.6400) and pericellular vGAT puncta with astrocytic processes (vGAT + S100B) (*P* = 0.7599) on PV neurons with PNN knockout using AAVSynCreGFP (*n* = 40 cells/4 mice per group). Bar represents mean ± s.d. Dots represent data points. One-way ANOVA, Tukey’s post hoc test (**c**) and unpaired two-tailed *t*-test (**e**,**g**,**i**,**l**,**n**,**o**,**q**,**s**,**t**,**w**,**y**,**za**). Scale bars, 2 µm (**h**,**j**,**m**,**r**,**v**,**w**,**x**,**z**). Both male and female mice were used. Illustrations in **a**,**u** created with BioRender. Source data

**Extended Data Fig. 5:**
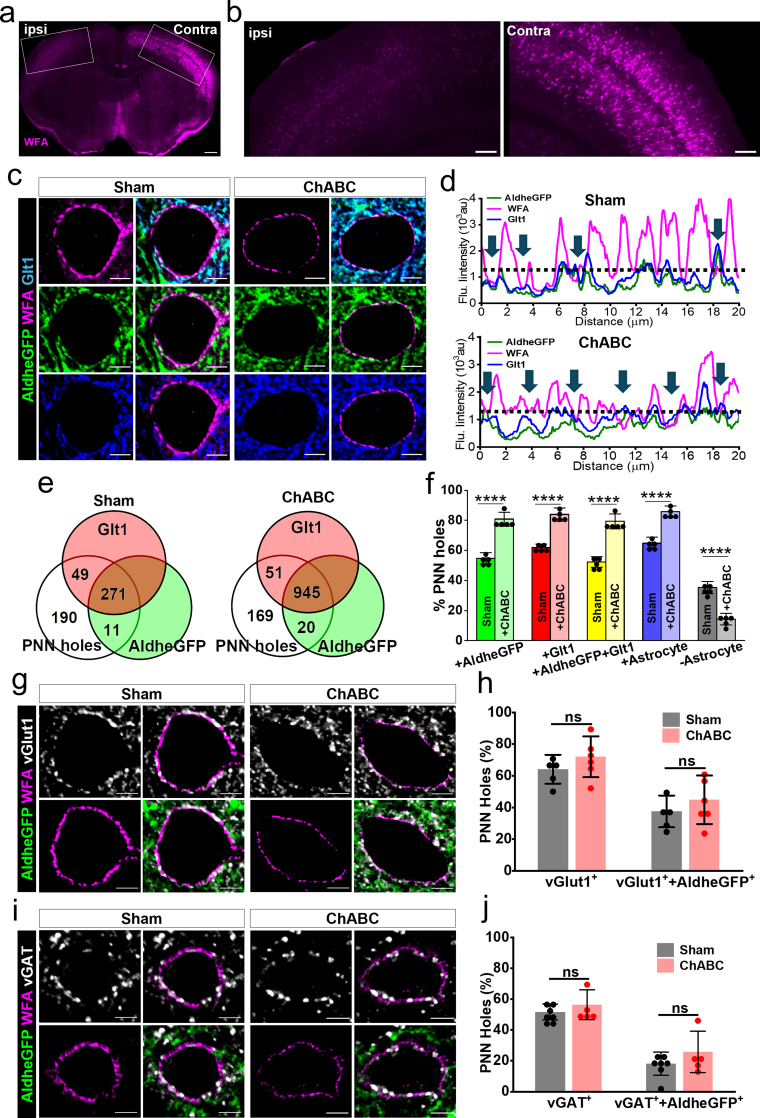
Astrocytic processes occupy newly formed PNN holes after ChABC treatment. **a-b**, IHC of PNN (WFA) in mouse brain (a) showing ChABC injected (ipsi) and contralateral (contra) hemispheres. Areas in boxes (a) are magnified in (b). Scale bar 500 µm (a), 200 µm (b). **c**, IHC of cortical PNN (WFA), and astrocytic markers (AldheGFP, Glut1) in sham and ChABC-injected mouse brains. **d**, Line intensity profiles of cortical PNN from sham and ChABC-injected brain showing high occupancy of PNN perforations with astrocytes (arrows). Dotted line represents WFA threshold. **e**, Proportional occupancy of cortical PNN holes by astrocytic processes in sham and ChABC-injected brains. **f**, Percent of total cortical PNN holes in sham and ChABC-injected brains occupied by AldheGFP (****P < 0.0001), Glt1 (****P < 0.0001) and both (****P < 0.0001), any astrocytic marker positive (****P < 0.0001) and any astrocytic marker negative (****P < 0.0001) holes. n = 40 PNNs/5 mice each group. One-way ANOVA, Tukey’s post-hoc test. **g**, IHC of cortical PNNs (WFA), astrocytes (AldheGFP), and excitatory synapses (vGlut1), in sham and ChABC-injected brains. **h**, Unaltered total pericellular vGlut1^+^ synapses (P = 0.2683) and pericellular vGlut1 synapses with astrocytic (vGlut1^+^+AldheGFP^+^) contacts (P = 0.3675) in cortical PNN holes in the ChABC-injected brains. n = 22PNNs/5 mice (sham), 35PNNs/6 mice (ChABC) per group. **i**, IHC of cortical PNN (WFA), astrocytes (AldheGFP), and inhibitory synapses (vGAT) from sham and ChABC-injected brains. **j**, Unaltered total pericellular vGAT synapses (P = 0.3614, n = 40 PNNs/7 mice (sham), 26PNNs/5 mice (ChABC)) and pericellular vGAT synapses with astrocytic contacts (P = 0.2922, n = 22PNNs/7 mice (sham), 35PNNs/5 mice (ChABC)) in cortical PNN holes in the ChABC-injected brains. Scale bar 5 µm in c, g, i. Unpaired two-tailed t-test in h and j. Bar data represent mean ± SD; dots represent data points. Source data

Supporting experience-dependent synaptic plasticity, PNNs are dynamic structures that undergo constant remodeling^6,19^, and in light of the above findings, should show plasticity regarding astrocytic cell coverage as well. We degraded cortical PNNs in vivo by intracranial injection of adult male and female Aldhe1l1-eGFP mice with chondroitinase ABC (ChABC). Comparison of 6-day post-ChABC injection with sham animals (Fig. 4a) shows a widespread decrease in WFA intensity (Fig. 4b,c) as well as increased perforations (Fig. 4d,e), leaving PNNs less dense and more porous (Extended Data Fig. 5a,b) with an increase in extracellular space in the pericellular region.

PNN depletion did not change the total astrocytic coverage as judged from Aldhe1l1-eGFP in a given field of view (Fig. 4f,g); however, the cell surface-associated pericellular astrocytic coverage on PNN-expressing neurons (PNN^+^) increased significantly (Fig. 4h–j) consistent with a change in coverage confined to the pericellular area previously occupied by the PNN. PNN-lacking neurons (PNN^−^) did not show any change in the pericellular coverage in the ChABC-treated condition (Fig. 4h,i) consistent with increased astrocytic coverage being due to the depletion of the PNN rather than disruption of diffuse chondroitin sulfate proteoglycans.

The increased pericellular astrocytic coverage following PNN disruption also significantly increased the number of astrocytic contacts on a now highly perforated PNN (Extended Data Fig. 5c–f). The astrocytic occupancy traced by GLT1 and/or AldheGFP markers (Extended Data Fig. 5c,d) increased from ~60–70% of PNN holes in controls (Fig. 1 and Extended Data Fig. 5e) to 80–90% (Extended Data Fig. 5e,f) in the ChABC-treated group.

While PNN disruption increased astrocytic coverage of neurons, it did not alter axosomatic synapses. ChABC treatment neither changed the total vGlut1 (Fig. 4k,l) nor the total vGAT terminals (Fig. 4p,q). Even when analysis was restricted to the pericellular area, in which astrocytic coverage was increased, there was no significant change in the densities of pericellular vGlut1 (Fig. 4m,n) and vGAT (Fig. 4r,s) contacts upon PNN disruption. We also assessed the pericellular vGlut1 (Fig. 4m,o) or vGAT (Fig. 4r,t) contacts closely associated with the astrocytic processes; however, no significant changes were observed in these groups. We also used the line intensity profile method to assess the occupancy of PNN holes in the ChABC-treated group (Extended Data Fig. 5c,d). Despite a significantly higher occupancy of PNN holes by astrocytic processes (Extended Data Fig. 5e,f), no significant changes were observed either in the occupancy of vGlut1 terminals or vGlut1 terminals with astrocytic contacts within PNN perforations (Extended Data Fig. 5g,h). Similarly, no significant differences were found in total vGAT terminals as well as in vGAT terminals in close association with astrocytes within PNN perforations (Extended Data Fig. 5i,j).

Together, in the adult mouse somatosensory cortex axosomatic synapses embedded in PNN holes are stable and resistant to degradation of PNNs, yet astrocytic processes are plastic and expand to occupy vacant territory on the neuronal surface.

### PNN deletion induces astrocytic plasticity without altering synapses

As transient PNN depletion using ChABC in adults caused increased astrocytic coverage occupying newly created perforations without changing abundance of presynaptic terminals, we sought to investigate whether a permanent genetic deletion of PNNs in the adult brain similarly destabilizes axosomatic synapses in conjunction with changing the pericellular astrocytic coverage.

PNNs were permanently depleted by intracranial injection of a viral vector carrying Cre recombinase with eGFP reporter (AAV9.hSyn.HI.eGFP.WPRE.SV40; AAV9.Cre) in the somatosensory cortex of adult male and female *Acan*^*fl/fl*^ mice (Fig. 4u) as described previously^20^. PNNs were eliminated after 8 weeks in all transduced PV neurons (Fig. 4u). As astrocytes in *Acan*^*fl/fl*^ lack eGFP in astrocytes, we used S100B, GLT1 and Kir4.1 in conjunction with PV and WFA to quantify the pericellular coverage of astrocytes upon PNN depletion (Fig. 4v). With all three markers the pericellular astrocytic coverage showed a consistent increase around PV neurons upon PNN deletion compared to neurons with intact PNNs (Fig. 4v,w); however, we did not observe any changes in pericellular density of vGlut1 terminals or vGlut1 terminals associated with S100B-expressing astrocytic processes (Fig. 4x,y). Similarly, pericellular density of vGAT-labeled inhibitory synaptic terminals or vGAT terminals associated with astrocytic processes (Fig. 4z,za) remained unaltered on PNN elimination.

These data suggest that both temporary or permanent degradation of PNNs in the adult brain induces similar astrocytic structural plasticity around PV neurons without changing the abundance of excitatory and inhibitory axosomatic synapses with and without astrocytic contacts. In conjunction with the developmental PNN deletion findings in Fig. 3, it can be concluded that developmental PNN deposition locks tripartite synapses and terminates astrocytic structural plasticity, which can be reinstated upon PNN depletion in adult brains; however synaptic contacts, once formed, are highly stable.

### PNN disruption in Alzheimer’s disease and epilepsy alters astrocytic coverage

**Fig. 5:**
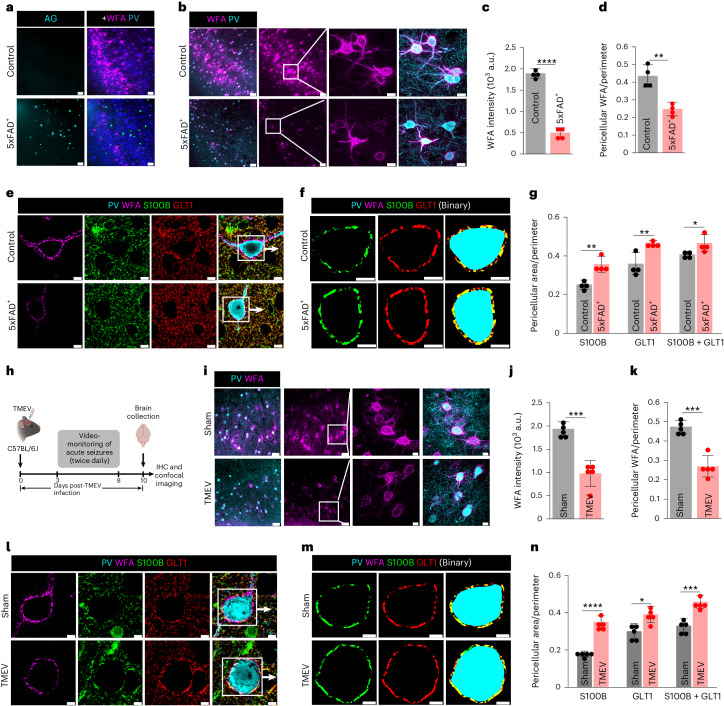
Disrupted PNN and increased astrocytic coverage in Alzheimer’s disease (5xFAD) and epilepsy (TMEV) mice models. **a**, IHC showing PNN loss (WFA) and amyloid plaques (Amylo-Glo, AG) in 5xFAD^+^ mice cortex. Scale bars, 50 µm. **b**, IHC showing altered abundance and 3D architecture of cortical PNN (WFA) in 12-month-old 5xFAD^+^ mice. Scale bars, 50 µm, magnified images 10 µm. **c**,**d**, Reduced cortical PNN (WFA) intensity (**c**) (****P < 0.0001) and PNN (WFA) coverage (**d**) (**P = 0.0039) in 5xFAD^+^ mice cortex. *n* = 4 mice per group in **c**,**d**. **e**,**f**, IHC (**e**) and binary images (**f**) of PNN (WFA) and astrocytic markers (S100B and GLT1) showing increased pericellular astrocytic coverage in 5xFAD^+^ mice cortex. Scale bars, 5 µm. **g**, Increased pericellular astrocytic coverage by astrocytic markers S100B (**P = 0.0043), GLT1 (**P = 0.0095) and combining both (S100B + GLT1) (*P = 0.0431) in 5xFAD^+^ mice cortex. *n* = 4 mice per group. **h**, Generation in TMEV model of acute seizures in mice. Illustration created using BioRender. **i**, IHC showing altered abundance and 3D architecture of cortical PNN (WFA) 10 days post-TMEV-induced seizure. Scale bars, 50 µm, magnified images 10 µm. **j**,**k**, Reduced cortical PNN (WFA) intensity (**j**) (***P = 0.0004), and PNN (WFA) coverage (**k**) (***P = 0.0002) in TMEV model of acute seizure. *n* = 5 mice per group. **l**,**m**, IHC (**l**), and binary images (**m**) of cortical PNN (WFA) and astrocytic markers (S100B and GLT1) showing increased pericellular astrocytic coverage in TMEV model of acute seizure. Scale bars, 5 µm. **n**, Increased pericellular astrocytic coverage by astrocytic markers S100B (****P < 0.0001), GLT1 (*P = 0.0107) and combining both (S100B + GLT1) (***P = 0.0005) in TMEV model of acute seizure. *n* = 5 mice per group. Bar represents mean ± s.d.; dots represent data points. NS, not significant, *P* > 0.05; unpaired two-tailed Student’s *t*-test (**c**,**d**,**g**,**j**,**k**,**n**). We used adult male and female mice. Source data

**Extended Data Fig. 6:**
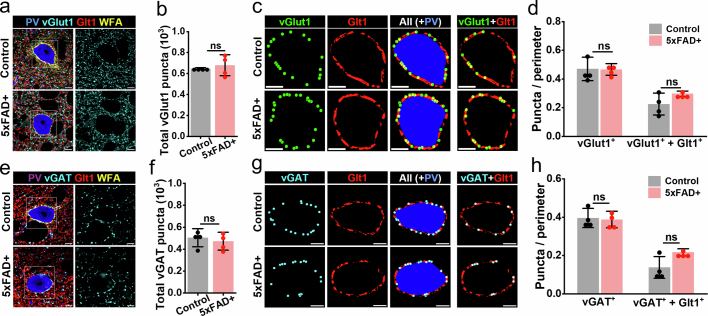
PNN disruption in 5xFAD model of Alzheimer’s disease without altered synaptic contacts. **a**, IHC showing cortical PNN (WFA), PV neuron, glutamatergic synapses (vGlut1), and astrocytic processes (Glt1), in control and 12-month old 5xFAD^+^ mice. **b**, Unaltered total vGlut1 puncta in 5xFAD^+^ mice cortex (P = 0.5669, n = 4 mice per group). **c**, Binary images showing total pericellular vGlut1 synapses, astrocytic coverage (Glt1) around cortical PV neurons in control and 5xFAD^+^ mice. The rightmost image shows vGlut1 synapses associated with astrocytic Glt1. **d**, Unaltered total pericellular vGlut1 synapses (P = 0.9376), and pericellular vGlut1 synapses associated with astrocytic Glt1 (P = 0.1537) in 5xFAD^+^ mice cortex. n = 4 mice per group. **e**, IHC showing cortical PNN (WFA), PV neuron, GABAergic synapses (vGAT), and astrocytic processes (Glt1), in control and 12-month old 5xFAD^+^ mice. **f**, Unaltered total vGAT synapses in 5xFAD^+^ mice cortex (P = 0.6001, n = 4 mice per group). **g**, Binary images showing total pericellular vGAT synapses, astrocytic coverage (Glt1) around cortical PV neurons in control and 5xFAD^+^ mice. Rightmost image shows vGAT synapses associated with astrocytic Glt1. **h**, Unaltered pericellular total vGAT synapses (P = 0.8158), and pericellular vGAT synapses associated with astrocytic Glt1 (P = 0.0632) in 5xFAD^+^ cortex. n = 4 mice per group. Unpaired two-tailed t-test in b, d, f, and h. Bar data represent mean ± SD; dots represent data points. Scale bar 5 µm in all. Source data

To question whether PNN disruption in the context of disease similarly influences astrocytes and tripartite synapses, we turned to the 5xFAD model of Alzheimer’s disease, and Theiler’s murine encephalomyelitis virus (TMEV) model of epilepsy. Male and female 5xFAD^+^ mice showed amyloid plaques (Fig. 5a) and widespread degradation of PNNs in the somatosensory cortex (Fig. 5b–d) at 12 months of age. Pericellular astrocytic coverage (S100B/GLT1) of PV neurons (PV/WFA) (Fig. 5e) was significantly higher in 5xFAD^+^ mice (Fig. 5f,g) yet the number and pericellular density of excitatory (Extended Data Fig. 6a–d) and inhibitory (Extended Data Fig. 6e–h) synapses with and without astrocytic contacts remained unchanged.

Epilepsy is another brain pathology in which PNN disruption has been observed^5,15^. Intracranial injection of TMEV into adult male and female C57BL/6 mice causes handling-induced seizures within 2–6 days after infection (Fig. 5h), followed by chronic spontaneous seizures after a variable epileptogenesis period and showed a widespread degradation of PNNs in the somatosensory cortex (Fig. 5i–k). S100B and GLT1-labeled astrocytic processes showed a significantly higher pericellular coverage around PV neurons in TMEV-injected mice (Fig. 5l–n).

These data suggest that PNN disruption in CNS pathologies is also associated with an expansion of astrocytic coverage; however, synaptic contacts remain largely stable.

### PNN facilitates astrocytic uptake of synaptically released Glu

Based on the 3D reconstruction analysis (Figs. 1 and 2), we hypothesized that PNNs around tripartite synapses may act as a diffusion barrier to contain synaptically released Glu and K^+^ for efficient uptake by astrocytes and hence loss of PNNs may impair their clearance by astrocytes.

**Fig. 6:**
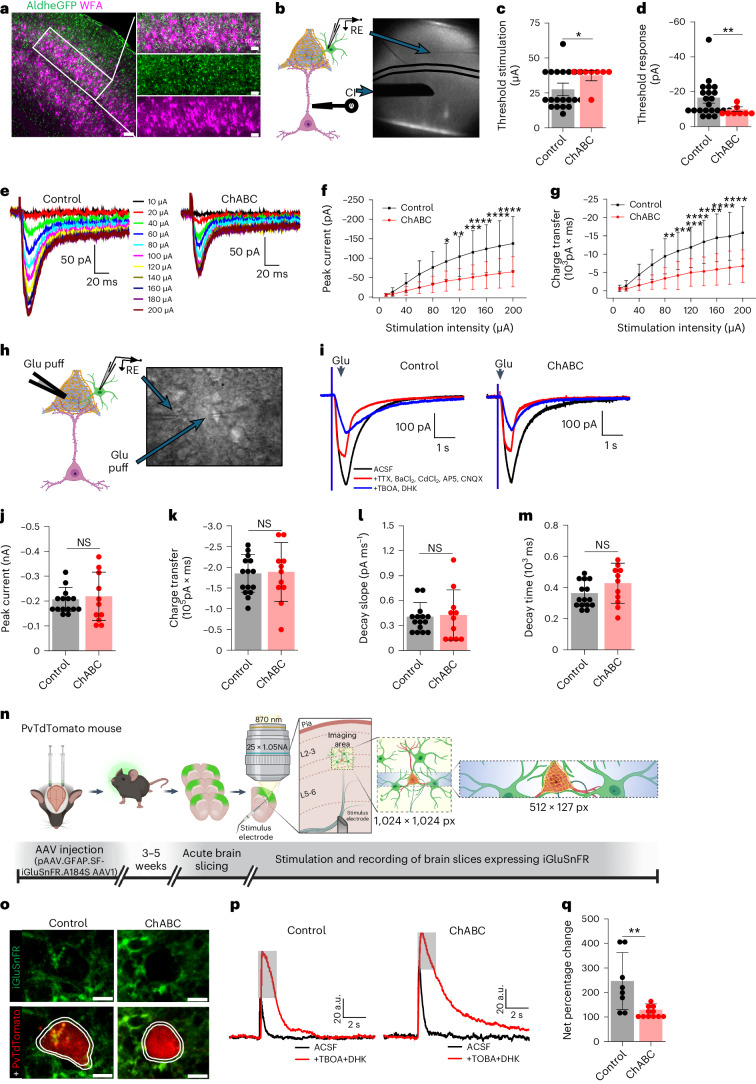
Acute cortical PNN depletion disrupts Glu uptake by astrocytes. **a**, IHC of cortical PNN (WFA) and astrocytes (AldheGFP) in L3–4 of SSC showing an average 50-µm diameter territory of individual astrocytes (circles in magnified image) encompasses one or more PNNs. Scale bars, 50 µm. **b**, Schematics and brightfield image showing current injector (CI) causing synaptic Glu release and subsequent recording of Glu uptake current by astrocytic processes in PNN holes. RE, recording electrode. **c**,**d**, Higher threshold stimulation (**c**) (*P = 0.0177, *n* = 20 cells/10 mice (control), 8 cells/5 mice (ChABC)) and lower uptake threshold response (**d**) (**P = 0.0082, *n* = 22 cells/10 mice (control), 8 cells/5 mice (ChABC)) of cortical astrocytes in ChABC-treated slices. **e**, Glu uptake current traces from cortical astrocytes in response to increasing stimulation intensity in control and ChABC-treated slices. **f**,**g**, Lower peak Glu uptake currents (**f**), and lower charge transfer (**g**) in cortical astrocytes in ChABC-treated slices. *n* = 24 cells/10 mice (control), 16 cells/6 mice (ChABC) in both **f** and **g**. ****P < 0.0001, ***P < 0.001, **P < 0.01, *P < 0.05 in **f** and **g**. **h**, Schematics and brightfield image showing Glu puff induced astrocytic Glu uptake current recording in cortical slice. **i**, Astrocytic Glu uptake currents on puffing 200 µM Glu in control and ChABC-treated cortical slice. **j**–**m**, Unchanged peak Glu current (*P* = 0.7071) (**j**), total charge transfer (*P* = 0.8837) (**k**), decay slope (*P* = 0.8405) (**l**) and current decay time (*P* = 0.1649) (**m**) of astrocytes in ChABC-treated slices. *n* = 15 cells/8 mice (control), 11cells/5 mice (ChABC) in **j**–**m**. **n**, Experimental schematics. **o**, Two-photon images of PV neuron (PvTdTomato) and pericellular astrocytic processes expressing iGluSnFR (iGluSnFR) in control and ChABC-treated cortical slice. Scale bars, 5 µm. **p**, iGluSnFR fluorescence traces from astrocytic processes (areas within lines (**o**)) on synaptic Glu release, before (ACSF) and after (+TBOA + DHK) blocking Glu transport in control and ChABC-treated cortical slices. Gray bar represents net fluorescence change, thereby net astrocytic uptake. **q**, Reduced net astrocytic Glu uptake in ChABC-treated cortical slices. **P = 0.0044, *n* = 8 recordings/7 mice (control), 11 recordings/7 mice (ChABC), unpaired two-tailed Student’s *t*-test (**c**,**d**,**j**–**m**,**q**) and two-way ANOVA, Tukey’s post hoc test (**f**,**g**). Bar data represent mean ± s.d. Dots represent data points. We used adult male and female mice. Illustrations in **b**,**h**,**n** were created using BioRender. Source data

**Extended Data Fig. 7:**
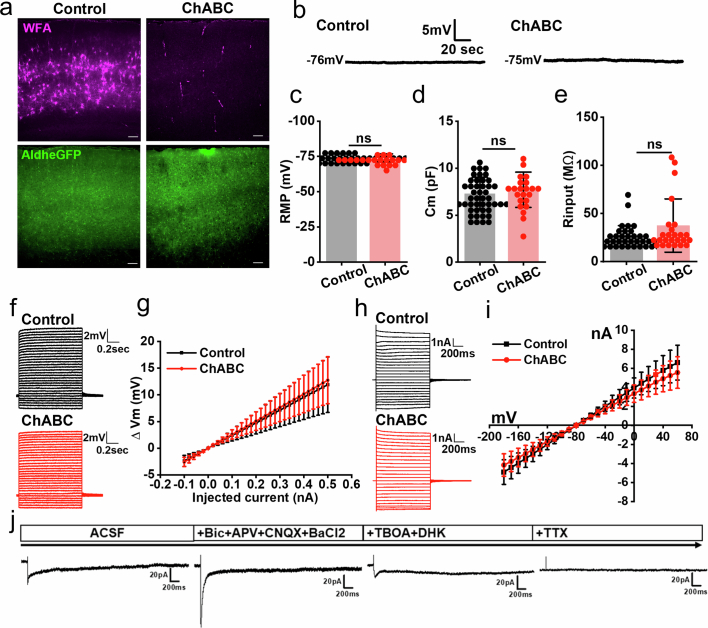
Biophysical properties of astrocytes remain unchanged on PNN disruption with ChABC. **a**, IHC of control and ChABC-treated acute cortical slices fixed and stained after electrophysiological recordings showing astrocytes (AldheGFP) and degraded PNN (WFA). Scale bar 100 µm. **b**, Membrane voltage traces of astrocytic resting membrane potential from control and ChABC-treated cortical slices. **c – e**, unchanged (**c**) resting membrane potential (P = 0.4996, n = 49c/8 m (control), 24c/ 6 m (ChABC)), (d) membrane capacitance (P = 0.3968, n = 46c/17 m (control), 22c/7 m (ChABC)), and (**e**) input resistance (P = 0.0646, n = 41c/18 m (control), 25c/7 m (ChABC)) of cortical astrocytes in ChABC-treated slices. Unpaired two-tailed student’s t-test in c - e. **f – g**, Current clamp traces (**f**) and IV plot (**g**) showing the current-voltage relationship of astrocytes in control (n = 49c/18 m) and ChABC-treated (n = 28c/9 m) slices. **h – i**, Representative voltage clamp traces (**h**), and IV plot (**i**) showing the current-voltage relationship of astrocytes in control (n = 59c/18 m) and ChABC-treated (n = 38c/8 m) cortical slices. **j**, Representative voltage clamp traces of synaptically evoked currents in astrocytes in presence of different blockers to isolate astrocytic Glu uptake current. c and m (c/m) represent number of cells and mice respectively. Bar data and error plots g and i represent mean ± SD. ns – not significant. Source data

Depletion of PNN with ChABC dissolved PNNs (Extended Data Fig. 7a) without altering biophysical properties of patch-clamped astrocytes as judged by undisturbed resting membrane potential (Extended Data Fig. 7b,c), low membrane capacitance (Extended Data Fig. 7d), input resistance (Extended Data Fig. 7e), and input–output curves (Extended Data Fig. 7f–i) in acute cortical slices from adult FVB-N//Swiss Webster-Aldh1l1-eGFP mice of both sexes. Synaptically evoked astrocytic Glu uptake currents (stimulation of L5–6 axonal fibers) were recorded in L3–4 astrocytes where each astrocyte contacts one or more PNNs (Fig. 6a,b). The distance between the stimulator and patch pipette was kept identical for all experiments (Fig. 6b). The astrocytic Glu transporter current was recorded in the presence of bicuculline, BaCl_2_, d-AP5 (d-2-amino-5-phosphonovalerate) and CNQX (6-cyano-7-nitroquinoxaline-2,3-dione) as described previously^21,22^ and isolated with a cocktail of TBOA (dl-threo-β-Benzyloxyaspartic acid) and DHK (dihydrokainic acid)^23–25^. The small fraction of remaining current was blocked by 0.5 µM tetrodotoxin (TTX) confirming that the recorded current was indeed the synaptically evoked glutamate uptake current (Extended Data Fig. 7j).

The minimum amount of current required (threshold stimulation) to generate a reliably detectable Glu uptake current was significantly higher (Fig. 6c); however, the threshold uptake current (threshold response) was significantly smaller in ChABC-treated slices (Fig. 6d).

We next generated input–output curves of astrocytic Glu uptake currents (Fig. 6e). We experimentally determined a near linear stimulus range from 10–200 µA with a 20 µA increment suitable for the input–output curve. ChABC-treated slices showed a significant decrease in the peak Glu uptake (Fig. 6f) as well as reduced total charge transfers (Fig. 6g) suggesting that the presence of intact PNNs yields an increase in astrocytic Glu uptake in response to synaptic activation.

**Extended Data Fig. 8:**
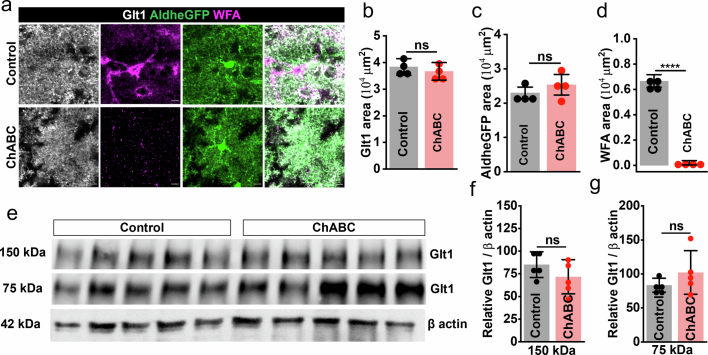
Unaltered glutamate transporter expression on PNN depletion in acute cortical slices. **a**, IHC of control and ChABC-treated acute cortical slices fixed and stained after electrophysiological recordings showing astrocytes (AldheGFP, Glt1) and degraded PNN (WFA). Scale bar 10 µm. **b**–**d**, Unaltered IHC expression of Glt1 (**b**) (P = 0.4895), AldheGFP (**c**) (P = 0.3516), and degradation of PNNs (**d**) (WFA) (****P < 0.0001) showing PNN disruption without altering astrocytic Glt1 expression. n = 4mice per group. **e**, Representative Western blot of cortical protein lysates from 5 control and 5 ChABC treated mice cortical slices showing Glt1 protein expression (∼150 kDa and ∼75 kDa) compared to the loading control β actin (∼42 KDa). Unprocessed blots are shown in supplementary material. **f-g**, Unaltered expression of ∼150 kDa (**f**) (P = 0.2449) and ∼75 kDa (**g**) (P = 0.2684) bands of Glt1 protein relative to β actin. n = 5 mice per group. Unpaired two-tailed student t-test in b - g. Bar data represent mean ± SD; dots represent data points. Source data

The reduced Glu uptake is not due to a nonspecific loss of astrocytic GLT1 transporter expression or function as exogenous Glu pulses delivered from a set distance to astrocytes (Fig. 6h), while blocking synaptic transmissions and other nonspecific currents as described previously^26^, produced identical Glu uptake current (Fig. 6i) and uptake kinetic (Fig. 6j–m) in controls and ChABC-treated cortical slices. Furthermore, GLT1 expression after ChABC digestion was unchanged as judged by IHC (Extended Data Fig. 8a–d) and western blot (Extended Data Fig. 8e–g).

Extensively arborized astrocytic processes contact multiple synapses including synapses on non-PNN-expressing neurons. Therefore, the above changes in astrocytic Glu uptake may be partly influenced by astrocytic processes encompassing synapses other than perisomatic synapses in the PNN holes. To specifically assess the Glu uptake by astrocytic processes in the pericellular area of PV neurons, we expressed the genetically encoded Glu sensor iGluSnFR^27^ in astrocytes using GFAP.SF.iGluSnFR.AAV1 in adult male and female PvTdTomato mice (Fig. 6n). A minimum 3-week incubation expressed iGluSnFR in astrocytic processes around the PV neurons in L3–4 of the somatosensory cortex (Fig. 6o) allowing Glu measurements on astrocytic processes in the pericellular area of PV neurons.

As with patch-clamp studies, we stimulated L5–6 axonal fibers and recorded iGluSnFR fluorescence from astrocytic processes encompassing a PV neuron in L3–4 (Fig. 6o). After examining a wide range of stimuli, we found that a single stimulation of 500 µA for 10 µs reliably generated >2 times higher signals than the minimum resolvable fluorescence change, therefore we used this as a standard stimulation paradigm for all experiments. We recorded iGluSnFR fluorescence intensity around PV neurons before (Fig. 6p, black traces ACSF) and after blocking astrocytic Glu transporters (Fig. 6p, red traces TBOA + DHK) and computed the net change in fluorescence intensity in control (Fig. 6p, gray bar and Supplementary Video 3) and ChABC-treated (Fig. 6p, gray bar and Supplementary Video 4) cortical slices as an indicator of net Glu uptake by astrocytes. The net change in iGluSnFR fluorescence was significantly lower in ChABC-treated slices compared to control (Fig. 6q) confirming that PNN disruption lowers the astrocytic Glu uptake at perisomatic tripartite synapses.

### PNN facilitates astrocytic uptake of depolarization released K^+^

**Fig. 7:**
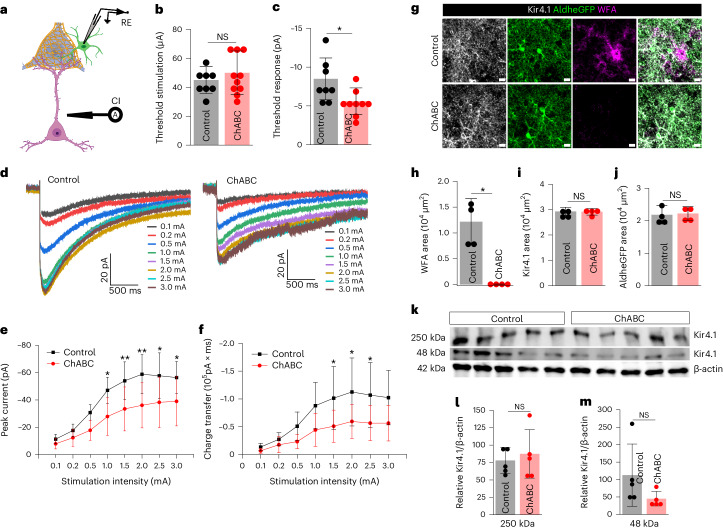
Acute cortical PNN digestion disrupts astrocytic K^+^ uptake. **a**, Schematics of synaptically evoked astrocytic K^+^ uptake current. **b**,**c**, Cortical astrocytes show unaltered threshold stimulation (**b**) (*P* = 0.3636, *n* = 8 cells/3 mice (control), 10 cells/5 mice (ChABC) and lower threshold K^+^ current response (**c**) (*P = 0.0248, *n* = 8 cells/3 mice (control), 9 cells/5 mice (ChABC) in ChABC-treated slices. **d**, K^+^ uptake current traces from cortical astrocytes in response to a series of increasing stimuli in control and ChABC-treated slices. **e**,**f**, Lower peak K^+^ uptake currents (**e**) (*n* = 11 cells/7 mice (control), 15 cells/6 mice (ChABC) and lower charge transfer (**f**) (*n* = 11 cells/7 mice (control), 13 cells/6 mice (ChABC)) in cortical astrocytes in ChABC-treated slices. **P < 0.01, *P < 0.05 (**e**,**f**). **g**, IHC of astrocytic proteins Kir4.1, AldheGFP and PNN (WFA) from fixed acute slices after ChABC treatment. Scale bar, 10 µm. **h**–**j**, IHC area quantification of PNN (WFA) (*P = 0.0123) (**h**), Kir4.1 (*P* = 0.8882) (**i**) and AldheGFP (*P* = 0.8333) (**j**) showing PNN disruption without changing astrocytic Kir4.1 expression. *n* = 4 mice per group. **k**, Western blot of cortical protein lysates from five control and five ChABC-treated mice brain slices showing Kir4.1 protein expression (∼250 kDa and ~48 kDa) compared to the loading control β-actin (~42 kDa). **l**,**m**, Unaltered expression of ∼250 kDa (*P* = 0.6137) (**l**) and ∼48 kDa (*P* = 0.1672) (**m**) bands of Kir4.1 protein relative to β-actin. *n* = 5 mice per group in **l**,**m**. Unprocessed blots are shown in Supplementary Fig. 3. Unpaired two-tailed Student’s *t*-test (**b**,**c**,**h**–**j**,**l**,**m**) and two-way ANOVA with Tukey’s post hoc test (**e**,**f**). Bar data represent mean ± s.d.; dots represent data points. We used adult male and female mice. Source data

Because the firing of fast-spiking interneurons enclosed by PNNs also release copious amounts of K^+^, we questioned whether PNN may also aid containment of K^+^ for astrocytic uptake. L5–6 stimulation induced K^+^ currents in L3–4 astrocytes in the presence of a cocktail blocking postsynaptic and astrocytic Glu currents (Fig. 7a). As with Glu uptake, astrocytic K^+^ uptake was also significantly attenuated on PNN disruption. Although astrocytes required similar magnitudes of threshold stimuli to evoke a detectable K^+^ current (Fig. 7b), the threshold response was significantly lower in ChABC-treated slices (Fig. 7c). Complementing the threshold response current, the input–output curve (Fig. 7d) of the synaptically evoked K^+^ currents showed a significantly lower K^+^ uptake current (Fig. 7e) resulting in a reduced total charge transfer (Fig. 7f). Neither Kir4.1 (Fig. 7g,i) nor AldheGFP (Fig. 7g,j) expression changed in the recorded slices upon ChABC treatment, which did eliminate PNNs (Fig. 7g,h). Corroborating IHC studies, western blot showed no significant difference in Kir4.1 expression after ChABC treatment of acute cortical slices (Fig. 7k–m) suggesting that altered K^+^ currents could not be attributed to a change in Kir4.1 expression in astrocytes.

Together, these data suggest that PNNs ensure the effective astrocytic clearance of synaptically released K^+^ and Glu; PNN disruption allows synaptically released molecules to diffuse into the extrasynaptic space.

### PNN disruption generates spontaneous seizures in mice

Impaired astrocytic clearance of Glu and K^+^ has been implicated in seizures^5,8^. We previously reported that an acute PNN disruption in ex vivo cortical slices facilitates hyperexcitability^15^ and other studies have shown a higher seizure susceptibility upon PNN degradation^28^. In addition, we report disruption of PNNs in 5xFAD model of Alzheimer’s disease and TMEV model of seizure and epilepsy. Hence one may speculate that the hyperexcitability associated with AD^29^ and epilepsy may be attributed to PNN disruption impairing astrocytic Glu and K^+^ uptake; however, the complexity of these diseases makes it difficult to assess the contribution of PNN disruption to neuronal hyperexcitability.

**Extended Data Fig. 9:**
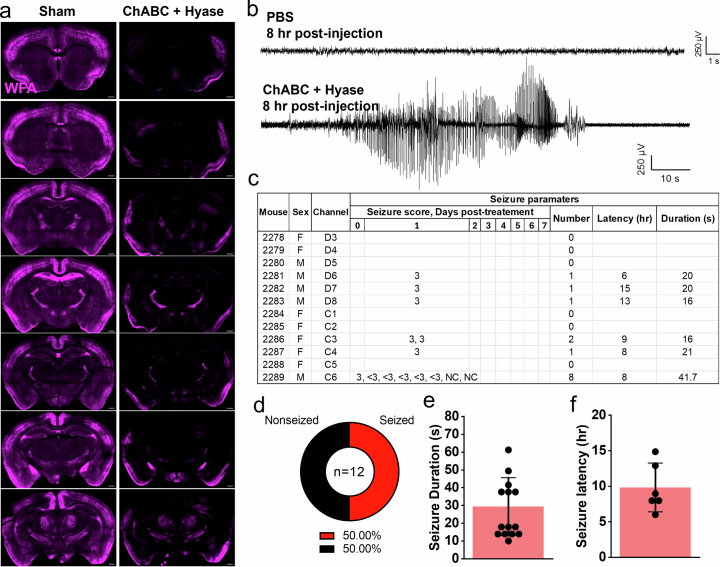
PNN disruption in-vivo generates seizures in mice. **a**, IHC showing PNN (WFA) in coronal brain sections along the rostro-caudal axis from PBS (sham) and ChABC + hyaluronidase (Hyase)-injected mice. Scale bar 500 µm. **b**, Representative cortical EEG traces showing normal activity in PBS injected mice (upper trace) and electrographic seizure in ChABC + Hyase injected mice (lower trace). **c**, Seizure score table. **d**, Pie chart showing seized and non-seized mice after ChABC + Hyase injections. **e – f**, Bar diagrams showing seizure durations (**e**) and seizure latency (**f**) in mice injected with ChABC + Hyase. n = 6 mice per group in both. Bar data shows mean ± SD; dots represent data points. Source data

As an alternative to study this hypothesis, we depleted PNNs in mice where we achieved a fast and widespread disruption of PNNs by bilaterally injecting a cocktail of ChABC and hyaluronidase in the rostral and caudal areas of the cortex (Extended Data Fig. 9a) of adult male and female FVB-N mice. We monitored the development of electrographic abnormalities and/or seizures by continuous video/electroencephalography (vEEG) comparing mice injected with ChABC plus hyaluronidase  with PBS-injected sham mice. Of the 12 injected mice, 6 exhibited robust electrographic and behavioral seizures (Extended Data Fig. 9b–d and Supplementary Video 5), ranging from 16–64-s duration (Extended Data Fig. 9e) with seizure latency ranging from 6–15 h. (Extended Data Fig. 9f). We likely missed seizures in some mice if they occurred during the first few hours post-injection as we did not record EEG during surgical recovery. The rapid onset of seizure activity in the injected mice strongly suggests that PNN degradation and the ensuing failure of astrocytic Glu and K^+^ uptake were causal in generating cortical hyperexcitability and consequent seizures.

## Discussion

Since Golgi’s description over a century ago, PNNs have fascinated neuroscientists. In the visual system, their role in stabilizing synapses is well documented. Those studies, however, were unaware that most excitatory and many inhibitory synapses are ensheathed by astrocytic processes and together form the tripartite synapse, where astrocytes support synaptic function through uptake of neuronally released transmitters and ions. Our objective was to question whether PNNs may be an important structural and functional component of the tripartite synapse.

We focused our work on cortical fast-spiking PV^+^ interneurons, as PNNs are predominantly expressed by them^30^. Our study describes the functional cooperation of PNNs and astrocytes in the clearance of neuronally released Glu and K^+^ at tripartite synapses. We find 90% of PNN holes contain either excitatory synapses, inhibitory synapses or both. About 70% of these synapses are tripartite, hence contain astrocytic leaflets. In all instances, these express Kir4.1, the astrocytic channel tasked with K^+^ uptake, as well as GLT1, the major excitatory amino acid transporter in astrocytes irrespective of whether they are associated with an excitatory or inhibitory synapse. Notably they do rarely harbor aquaporins, the water channels abundantly expressed in conjunction with Kir4.1 channels on astrocytic endfeet touching blood vessels. Using transient enzymatic or permanent genetic disruption of the PNNs we found that in the adult cortex, synapses on PV neurons are static and do not change upon PNN removal; however, astrocytic processes are plastic, and upon PNN removal astrocytes expand their territory on the cell soma. Astrocytes take possession of any membrane that was previously occupied by PNNs. This is different from the developing cortex where PNN deletion not only failed to restrict astrocytic plasticity but also increased the perisomatic synaptic contacts on PV neurons. From a pathological perspective, we find similar changes in astrocytic coverage and pericellular tripartite synapses associated with a natural PNN disruption occurring in mouse models of Alzheimer’s disease and epilepsy.

Mechanistically, we speculate that a wide range of signaling and/or cell adhesion molecules released or activated upon PNN disruption including Glu^12,31^, calcium^31^, neuroligins^31^, hyaluronan^32–34^, integrins and CD44 (refs. ^32–34^) can serve as putative messengers to trigger astrocytic cytoskeletal remodeling pathways including JAK–STAT3 and Rho GTPase^35^ specially REC1 (refs. ^32,34^) and Rho-associated kinase (ROCK), to effectuate subtle changes in astrocytic processes.

While the structural interactions of synapses and astrocytes and astrocyte plasticity are fascinating, we also uncovered an important functional synergy between PNNs and astrocytes. Having synapses embedded in densely packed PNNs contains ions and neurotransmitters in the local PNN pocket where astrocytic leaflets can effectively remove them. Not so, when the PNNs are disrupted and Glu spills out of the synapses as evident from increased [Glu] in the perisynaptic space. As PNNs are known to be degraded by proteolytic enzymes, including matrix metalloproteinases (MMPs) and A disintegrin and metalloproteinase with thrombospondin motifs (ADAMTSs)^6^, it is easy to envision how brain inflammation in the context of injury or disease will break down PNNs allowing spillage of K^+^ and Glu into the perisynaptic space. Increases in K^+^ and Glu are known contributors to epilepsy^8^ and inhibition of PNN proteolysis can suppress seizures^15^. Enzymatic PNN degradation causes epileptiform activity^36^, decreased seizure threshold/latency^15^ and exacerbated seizures^28,37,38^, and genetic disruption of PNNs or its components largely phenocopies such changes^39–41^. Our findings that spontaneous seizures develop upon PNN disruption in vivo support the postulated causal association between seizures and PNN disruption.

Activation of extrasynaptic NMDAR by spillage of Glu can induce neuronal death via activation of the p38–MAPK pathway^4^. The containment of axosomatic synapses on PV interneurons may be a protective strategy of particular importance for this group of fast-firing neurons. Capable of firing hundreds of action potentials per second, they release vast amounts of K^+^. PNNs may have evolved specifically to contain ions and neurotransmitters that are associated with rapid firing in the close vicinity of astrocytes. We recently showed that PNNs around cortical PV neurons also change the dielectric properties of the cell membrane such as to reduce the effective membrane capacitance, thereby facilitating burst firing of these neurons. We show that degradation of PNNs by MMPs, released from glioma, degrades PNNs, increases the membrane capacitance and impairs fast-burst firing^15^.

The high negative charge density of the ECM and PNNs has been proposed to interact electrostatically with extracellular cations thereby differentially affecting their diffusion from anions; however, such electrostatic interactions and consequent diffusion hindrance are plausible only at low ionic strength^42–44^, whereas cerebrospinal fluid (CSF) likely saturates the negative charges of PNNs rendering it electrostatically neutral. Our empirical findings suggest that PNNs act as a physical diffusion barrier without discriminating between cations and anions.

The finding that PNN degradation impairs Glu uptake and K^+^ buffering by astrocytes at tripartite synapses may have broader implications relevant to numerous acute and chronic neurological conditions. In addition to the obvious contribution to seizures, known to cause the release of MMPs^45,46^, these findings may also explain the hyperexcitability associated with Alzheimer’s disease. Moreover, changes in PV interneuron firing have been reported in the PFC in the context of schizophrenia^47^, and in the substantia nigra the release of dopamine comes from fast-spiking interneurons that may be negatively affected in Parkinson’s disease^48,49^. It is possible that in these diseases and others, loss of PNN integrity may also affect the ability of astrocytes to support effective clearance of neuronally released K^+^ and Glu. The idea that synaptic function, and not just structure, critically depends on the functional contribution of astrocytes working synergistically with the PNN, dubbed the ‘tetrapartite synapse’^50^, is an exciting concept that warrants further study.

## Methods

### Animals

All animal procedures were approved and performed following the ethical guidelines set by the University of Virginia Institutional Animal Care and Use Committee (IACUC). Mice were housed in groups of five in a facility in a 12-h light–dark cycle with controlled temperature (21 ± 1.5 °C) and humidity (50 ± 10%) and had access to food and water ad libitum. Aldhe1l1-eGFP (GENSAT project) mice expressing eGFP under astrocyte-specific promotor AldheGFP were generated as described previously^24^ and housed and bred according to IACUC guidelines. We received C57BL/6N-Acan^tm1c(EUCOMM)Hmgu^/H (European Mouse Mutant Archive stock EM:10224) from EUCOMM (UK Research & Innovation, Mary Lyon Center). The heterozygous mice were bred together to generate *Acan*^*fl/fl*^ mice. To generate brain-wide developmental PNN KO, *Acan*^*fl/fl*^ mice were bred with Nestin-Cre (B6.Cg-Tg(*Nes-cre*)1Kln/J, strain 003771-JAX) and confirmed by genotyping. PvTdTomato mice (C57BL/6-Tg (Pvalb-tdTomato) 15Gfng/J, strain 027395-JAX) and 5xFAD model mice of Alzheimer’s disease (B6SJL-Tg (APPSwFlLon,PSEN1*M146L*L286V) 6799Vas/Mmjax, strain 034840-JAX), FVB/NJ (strain 001800-JAX) and C57BL/6J (strain 000664-JAX) were purchased from The Jackson Laboratory and subsequently bred in an animal facility to generate experimental mice. We used adult 7–15-week-old mice of both sexes unless stated otherwise. All mice were genotyped to confirm the transgene expression or knockout before experimental use.

### Intracranial surgeries and injections

#### ChABC injection

Chondroitinase ABC from *Proteus* *vulgaris* (C3667-10UN, Sigma-Aldrich) was dissolved in sterilized PBS (50 mU µl^−1^); subsequently, 2 µl solution was injected unilaterally at an infusion rate of 200 nl min^−1^. Mice were anesthetized with 2–5% isoflurane and fixed to a stereotaxic apparatus (Leica Angleone stereotaxic model, 39464710) followed by a midline scalp incision and a 0.5-mm burr hole 2.0 mm lateral and 1.0 mm ventral to bregma and infused ChABC at ~2.0-mm deep from the cortical surface using a 10-μl syringe (World Precision Instruments, SGE010RNS). Sham control mice were injected with sterile PBS with an identical procedure. Mice were dosed with buprenorphine/Rimadyl and allowed to recover on a heating pad until mobile and were monitored daily for up to 5 days after surgery. Body weight was measured for 3 consecutive days after surgery and all mice were perfused on day 6 after injection. Similar to previous studies^32^, we also found that ChABC injection homogeneously digests PNNs; however, faint and granular remains of the digested PNNs could still be visualized (Extended Data Fig. 5a,b) on intensity-enhanced images to identify previously intact PNNs.

#### AAV injection and Acan knockout

To knock out PNN in adult mice brains, we injected pENN.AAV.hSyn.HI.eGFP-Cre.WPRE.SV40 (Addgene, 105540-AAV9) in 7–8-week-old *Acan*^*fl/fl*^ mice. In brief, AAV9 (2.7 × 10^13^ vg per ml) was diluted in PBS to achieve 1 × 10^13^ vg per ml concentration and 1.5 µl was injected in each hemisphere (from bregma: 0.5 mm posterior, 2.0 mm lateral and 1.0 mm ventral) with 200 nl min^−1^ infusion rate as described above. Mice were transcardially perfused after 8–10 weeks of AAV9 injections to perform IHC.

#### AAV injection and iGluSnFR expression in astrocytes

Male and female PvTdTomato mice (6–12-week-old) were intracranially injected (2.0 mm lateral and 0.25 mm caudal from bregma on both hemispheres) with iGluSnFR AAV (pAAV.GFAP.SF-iGluSnFR.A184S AAV1, Addgene 106192-AAV1). Then, 1.20 µl of iGluSnFR AAV (1.6 × 10^13^ genome copies per ml) was injected at a rate of 0.12–0.15 µl min^−1^ using a 10-µl syringe (Hamilton, CAL800000 1701N). Mice were dosed with buprenorphine/Rimadyl and allowed to recover on a heating pad until mobile and were monitored daily for up to 5 days post-surgery. All mice receiving AAV injections were used for experiments within 3–5 weeks of AAV injection.

### Acute slice electrophysiology

Whole-cell patch-clamp recordings were obtained from astrocytes in situ acute brain slices as described previously^21^. In brief, mice underwent cervical dislocation followed by a quick decapitation and dissection to remove brains and were kept in an ice-cold ACSF (135 mM NMDG, 1.5 mM KCl, 1.5 mM KH_2_PO_4_, 23 mM choline bicarbonate, 25 mM d-glucose, 0.5 mM CaCl_2_ and 3.5 mM MgSO_4_, pH 7.35, 310 ± 5 mOsm) (all from Sigma-Aldrich) saturated with carbogen (95% O_2_ + 5% CO_2_). We prepared 300-μm thick coronal slices using Leica VT 1000P or 1200S tissue slicers. Slices were transferred into a custom-made recovery chamber filled with ACSF (125 mM NaCl, 3 mM KCl, 1.25 mM NaH_2_PO_4_, 25 mM NaHCO_3_, 2 mM CaCl_2_, 1.3 mM MgSO_4_ and 25 mM glucose, pH 7.35, 310 ± 5 mOsm) constantly bubbled with carbogen (95% CO_2_ + 5% O_2_) to recover at 32 °C for 1 h. Subsequently, slices were transferred to room temperature conditions until used for recordings. Individual slices were transferred to a recording chamber that was continuously superfused with ACSF at a flow rate of 2 ml min^−1^. GFP-positive astrocytes in Aldh1l1-eGFP mice cortical slices were visualized using an upright microscope (Leica DMLFSA) with ×5 and ×40 water-immersion lens and epifluorescence and infrared illuminations to identify eGFP-expressing astrocytes.

Whole-cell voltage-clamp and current-clamp recordings were conducted using an Axopatch 200B amplifier (Molecular Devices) with an Axon Digidata 1550A interface (molecular devices). Patch pipettes of 7–10 MΩ open-tip resistance were created from standard borosilicate capillaries (WPI, 4IN THINWALL Gl 1.5 OD/1.12 ID) using HEKA PIP 6 (HEKA) or PMP-102 (Warner Instruments) programmable pipette pullers. We filled patch pipettes with an intracellular solution containing 134 mM potassium gluconate, 1 mM KCl, 10 mM 4-(2-hydroxyethyl)-1-piperazineethanesulfonic acid (HEPES), 2 mM adenosine 5′-triphosphate magnesium salt (Mg-ATP), 0.2 mM guanosine 5′-triphosphate sodium salt (Na-GTP) and 0.5 mM ethylene glycol tetraacetic acid (EGTA) (pH 7.4, 290–295 mOsm). MM-225 micromanipulator (Sutter Instrument Co.) was used to visually guide the patch pipette to the cell. After making a tight seal of >5 GΩ resistance, brief suction was applied to achieve the whole-cell mode and cells were immediately clamped at −80 mV. The membrane capacitance (*C*_m_) and series resistance were not compensated. Data were acquired using Clampex 10.4 software and Axon Digidata 1550A interface (Molecular Devices), filtered at 5 kHz, digitized at 10–20 kHz and analyzed using Clampfit 10.6 or Clampfit 11.2 software (Molecular Devices). Unless otherwise stated, throughout all the recordings carbogen-bubbled ACSF was continuously superfused (2 ml min^−1^) and the bath temperature inside the reordering chamber was maintained at 32–33 °C using an inline feedback heating system (TC 324B, Warner Instruments).

#### PNN degradation in ex situ brain slices

ChABC from *Proteus* *vulgaris* (C3667, Sigma-Aldrich) was reconstituted in 0.01% bovine serum albumin aqueous solution according to the manufacturer’s instruction to make a 1 U per 40 μl stock solution. Aliquots of 1 U were prepared and stored at −20 °C until used. After slice recovery, slices were treated with ChABC and subsequent recordings were made as previously described^14,15^. In brief, after recovery, 2–3 cortical half slices were incubated in 3 ml ChABC solution (0.5 U ChABC per ml in ACSF) in an incubation chamber continuously supplied with carbogen at 33 °C for 45 min. Next, slices were rinsed twice and incubated in ACSF until used for electrophysiological recordings. These parameters of PNNs digestion by ChABC (enzyme concentration of 0.5 U ml^−1^, incubation time of 45 min and incubation temperature of 33 °C) reliably degraded PNNs (Extended Data Fig. 7a) as described previously^14,15^. For controls, previously separated contralateral halves of the ChABC-treated slices were incubated in 3 ml ACSF without ChABC and subsequently, both ChABC-treated and non-treated slices were kept in ACSF together until used for the recordings.

#### Measurement of intrinsic biophysical properties of astrocytes

The resting membrane potential (*V*_m_) was measured by setting *I* = 0 mode immediately after achieving the whole-cell configuration. The *C*_m_ was measured directly from the amplifier by adjusting capacitance and monitoring the capacitive transients as described previously^21^. To calculate the input resistance (*R*_in_) of astrocytes, we calculated the steady-stated membrane voltage deflection (Δ*V*) on injecting 15 hyperpolarization current steps (−100 pA each for 1,000 ms). The ratio (Δ*V*/*I*) of steady-state change in the membrane voltage (Δ*V*) and the corresponding injected current (*I*) was computed as *R*_in_. The *I*–*V* curve of astrocytes was computed in both the current clamp (31 steps, −100 pA to +500 pA, step size 20 pA, step duration 1,100 ms) and voltage clamp (25 sweeps, −180 mV to +60 mV, step size 10 mV) modes (Extended Data Fig. 7f–i) followed by plotting the voltage/current responses. Astrocytes with nonlinear *I*–*V* responses were not continued for recordings and analysis.

### Measurement of astrocytic currents

#### Synaptically evoked Glu uptake current

We recorded synaptically evoked currents from cortical astrocytes according to the previously published studies with some modifications^23^. In brief, we placed a concentric bipolar electrode (FHC, CBABD75) in L5–6 of the cortical slices and patched astrocytes in L3–4 of the somatosensory area (Fig. 6b). The stimulation protocol consists of initial 10-µA and 20-µA pulses followed by a 20-µA increment in each subsequent pulse capping at 200 µA (pulse duration 200 µs). All recordings were performed in the presence of 20 µM bicuculline, 50 μM d-AP5, 20 μM CNQX and 100 μM BaCl_2_. In the initial few recordings, we confirm that the recorded current is glutamate by observing a near-complete blockade of evoked current upon 100 μM TBOA and 300 μM DHK application (Extended Data Fig. 7j). The remaining current was abolished by superfusing 0.5 μM TTX, confirming it as a neuronal-evoked Glu current (Extended Data Fig. 7j). Each stimulation pulse was repeated five times (sweeps) and a minimum of two sweeps were averaged to compute the peak current and charge transfer after excluding the sweeps with baseline fluctuation or noise.

#### Depolarization evoked potassium uptake current

To record depolarization evoked astrocytic potassium uptake current, we positioned the stimulator and patch pipette as described above and incubated slices in a mixture of 20 µM bicuculline, 50 μM d-AP5, 20 μM CNQX, 100 μM TBOA and 300 μM DHK. The stimulation protocol consisted of initial 0.1-mA and 0.2-mA pulses followed by 0.5 mA and five subsequent pulses with 0.5-mA increments capping at 3 mA (pulse duration 200 µs). Each stimulation pulse was repeated three times (sweeps) and a minimum of two sweeps were averaged to compute the peak current and charge transfer after excluding the sweeps with baseline fluctuation or noise.

#### Astrocytic uptake of exogenously applied Glu

To measure the Glu uptake capacity of astrocytes we adopted the exogenous Glu puffing method as described previously with minor modifications^21,24^. In brief, we constantly perfused slices with ACSF containing 500 nM TTX, 20 μM bicuculline, 100 μM CdCl_2_, 50 μM d-AP5 and 50 μM CNQX and 100 μM BaCl_2_. After patching an astrocyte, a 500-ms puff (2 psi pressure using a Pico-liter Injector PLI-10 from Warner Instruments) of 200 μM glutamate solution (120 mM NaCl, 3.5 mM KCl, 25 mM HEPES, 10 mM glucose and 200 µM Glu) was applied from a distance of ~100 μm by a 5–8 MΩ open-tip resistance glass pipette. In several random recordings, we applied a mixture of 100 μM TBOA and 300 μM DHK to confirm that the recorded current was glutamate (Fig. 6i). We recorded five sweeps and averaged a minimum of three sweeps to generate a result sweep that was utilized to compute the data. The sweeps with fluctuating baseline and noise were excluded from the analysis. The averaged trace of uptake current was analyzed using Clampfit 10.6 or Clampfit 11.2 program to generate the below-described measurements. The peak current was calculated by subtracting the baseline from the peak response. The total charge transfer was computed by calculating the total areas under the curve of Glu uptake current response. Decay time and decay slope were calculated from the decaying phase (100% to 37% of the peak) of the uptake current.

### Two-photon microscopy imaging of astrocytic glutamate uptake using iGluSnFR

For live imaging of iGluSnFR in two-photon microscopy, recovered slices were placed in a recording chamber with continuous superfusion of carbogen-saturated ACSF at 2–3 ml min^−1^ with bath temperature maintained at 32–33 °C with an inline heater. Images were captured using a four-channel Olympus Dual-beam FVMPE-RS multiphoton microscope (Olympus/Evident) equipped with two high-sensitivity cooled GaAsP detectors, two multialkali photomultiplier detectors, a high-resolution Galvo and a fast resonant scanner, an XLPLN ×25/1.05 NA water-immersion objective (Olympus/Evident) and an InSight X3 ultrafast pulsed laser (Spectra Physics) with tuning range of 680–1,300 nm. For all experiments, the InSight laser was tuned to 870 nm for simultaneous excitation of both iGluSnFR and TdTomato. Olympus Fluoview software (Olympus/Evident) was used for all data acquisition and in combination with CellSens (Olympus/Evident) and Clampfit (Molecular Devices) software programs for subsequent analysis.

Slices were placed with the ventral side facing the field stimulating electrode and observed through the epifluorescence microscope to confirm TdTomato expression of PV cells (555 nm) and iGluSnFR fluorescence (488 nm). Areas of recording were determined by the presence of a PvTdTomato^+^ PV neuron with astrocyte presence in the immediate periphery/boundary of the cell. Single-frame images of the cells were taken using the high-resolution Galvo scanner at 1,024 × 1,024 pixels with a zoom of ×6. Imaging of realtime stimulation and subsequent changes of iGluSnFR expression was performed using the fast resonant scanner in a bidirectional scanning mode, imaging a region of 512 × 127 pixels with a zoom of ×6 resulting in an acquisition rate of approximately 40 Hz.

Synaptic release of Glu was achieved using a constant current isolated stimulator model DS3 (Digitimer) to emit a single pulse of current from the paired field stimulation electrode. Placement of the electrode consisted of locating the strongest expression of fluorescence in cortical L3–4 of the SSC region and tracing the neurites down to the corresponding cortical L5–6. Upon identification of a position with good fluorescence in both L3–4 and L5–6, the electrode was lowered into the bath and placed at the L5–6 location with very slight embedding into the surface of the slice. Following the placement of the electrode, the field of view was moved back up to the corresponding L3–4 of the SSC for visualization and recording of the fluorescent signal. A waveform generator (Olympus/Evident) was used to trigger the stimulation at an identical timepoint for each recording. To reliably detect the signal, we injected longer pulses of field stimuli than in previously described electrophysiology experiments, using stimulations of 10 ms of 500 μA.

All recordings consist of a brief baseline period followed by stimulation and finally a brief baseline recovery period. All recordings were performed first in ACSF followed by perfusing TBOA and DHK for 5–7 min and a repeated recording with TBOA and DHK in the bath. To minimize photobleaching and subsequent errors, we shortened the recording period and corrected the baseline using automated functions in the Clampfit program. We computed the peak intensities using the Clampfit program followed by subtracting the peak intensity before (ACSF) from that obtained after TBOA + DHK incubation (TBOA + DHK) to obtain the net change ((TBOA + DHK) − ACSF). To account for the baseline variability in fluorescence intensity, we used the peak intensity before TBOA + DHK (ACSF) as 100% and normalized the peak response after TBOA + DHK incubation (TBOA + DHK) and generated % net change (normalized to 100). We computed the % net change from control and ChABC-digested groups and performed statistical analysis.

### Seizure induction and EEG recordings

#### TMEV-induced seizures

We used Daniel’s strain of TMEV to induce seizures in mice. TMEV was provided by the laboratories of K. S. Wilcox and R. S. Fujinami from the University of Utah. The titer of the stock used was 2 × 10^7^ plaque-forming units (p.f.u.) per ml. Anesthetized mice (3% isoflurane) were injected with 20 µl of either PBS or TMEV solution (2 × 10^5^ p.f.u.) intracortically by inserting a 28-gauge needle perpendicular to the skull surface, slightly medial to the equidistant point on the imaginary line connecting the right eye and the right ear. We used a sterilized syringe containing a plastic sleeve on the needle to expose only 2.5 mm of the needle from the tip to restrict the injection in a cortical region. The needle was slowly retracted after 1 min of injection followed by disinfecting the injection site. Mice started behaving normally after 5–10 min of the injection.

To assess the handling-induced acute behavioral seizures between 2–8 days post-injection, we briefly agitated the cage by shaking and observed mice behavior for about 5 min. Seizures occurred within 1 min of handling and the seizure score was assessed using a modified Racine scale with stage 1 (mouth and facial movements); stage 2 (head nodding); stage 3 (forelimb clonus); stage 4 (forelimb clonus and rearing); stage 5 (forelimb clonus, rearing and falling); and stage 6 (intense running, jumping, repeated falling and severe clonus^18^).

#### PNN disruption and EEG electrode implantation surgery

A mixture of ChABC (C3667-10UN, Millipore Sigma) and hyaluronidase (H1136-1AMP, Millipore Sigma) enzymes was injected intracortically to degrade ECM in mice (FVB/N, The Jackson Laboratory; 8 weeks old). Stock solutions of ChABC (0.05 U µl^−1^) and hyaluronidase (2 U µl^−1^) were prepared by reconstituting lyophilized enzyme powder in sterile saline. The stocks were aliquoted in a single-use amounts (10 µl) to avoid thaw–freeze cycles and stored at −80 °C.

Mice were anesthetized using 3% isoflurane, provided analgesia (0.1 mg kg^−1^ buprenorphine and 5 mg kg^−1^ carprofen intraperitoneally) and heads were affixed into a stereotaxic instrument (David Kopf Instruments). The hair over the skull area was removed using a hair-removal cream, the surgical area was disinfected using iodine and 70% alcohol and the skull was exposed. Mice were continuously anesthetized using nasal tubing supplying 1–2% isoflurane throughout the surgical procedure. The aliquots of ChABC and hyaluronidase were thawed on ice just before use. An injection mixture of ChABC, hyaluronidase and saline was prepared in a ratio of 2:2:1 (10 µl ChABC + 10 µl hyaluronidase + 5 µl saline) sufficient for two mice. The enzyme preparations were kept on ice throughout the procedure. The control group of mice received saline injections. Two injection holes were drilled in the skull (−1.0 mm ML (mediolateral) from bregma, 1.0 mm or −2.0 mm AP (anteroposterior) from bregma). The enzyme mixture was then injected stereotactically into the cortex by inserting the needle into the brain at 45° angle to target the injections at −2.0 mm ML from bregma, 1.0 mm or −2.0 mm AP from bregma, and −0.5 DV (dorsoventral) from the brain surface. Neuros syringe (Model 1701, 65460-06, Hamilton) and 33-gauge needle (65461-02, Hamilton) were used to inject 4.5 µl solution per injection site at 0.6 µl min^−1^ injection speed (Quintessential Stereotaxic Injector, 53311, Stoelting). The needle was checked for any blockage by pumping a small amount of fluid just before inserting it into the brain. The needle was kept undisturbed for 2 min after injection and slowly retracted to prevent leakage of any fluid. The holes in the skull were filled with bone wax. With this procedure, a single mouse received a total of 0.18 U of ChABC and 7.2 U hyaluronidase in the left cortex.

To implant electrodes for the EEG recordings, a total of six holes (three for anchor screws and three for electrodes) were drilled in the skull carefully without causing bleeding. Two electrodes of a three-channel electrode set up (MS333/8-A, P1 Technologies) were implanted in the cortex bilaterally using stereotaxic coordinates of ±2.0 mm lateral and 1.5 mm posterior from bregma and about 0.5 mm ventral from the brain surface. The ground electrode was implanted over the brain surface near the cerebellum (−1.0 mm lateral and 5.0 mm posterior from bregma). Three anchor screws were carefully inserted into the skull without damaging the brain surface; the first one anterior to the bregma in the right hemisphere, the second one over the left parietal cortex and the third one posterior to the lambda in the right hemisphere. The electrodes and screws were secured in position using dental cement and the skin incision was closed using tissue glue. All surgically operated mice were treated humanely and provided with postoperative care as per the National Institutes of Health guidelines and the IACUC protocol.

#### Video EEG acquisition and seizure analysis

The vEEG was initiated after 3 h of surgical procedure and acquired continuously for 7 days. Mice were connected to an EEG100C differential amplifier (BIOPAC Systems) using a lightweight three-channel cable with a three-channel rotating commutator (P1 Technologies). The MP160 data acquisition system and AcqKnowledge 5.0 software (BIOPAC Systems) were used to record electroencephalograms. M1065-L network camera (Axis Communications) and media Recorder 4.0 software (Noldus Information Technology) were used to record the behavior of each mouse. All the cables and electrical components were sufficiently shielded to minimize electrical noise. Video and EEG recordings were automatically synchronized using Observer XT 14.1 software (Noldus Information Technology). EEG signals were bandpass filtered between 0.5 and 100 Hz, amplified and digited at a sampling frequency of 500 Hz. Mice had access to food and water conveniently during the entire vEEG recording.

The EEG and video recordings were reviewed manually by an experimenter blinded to the treatment group of mice. Electrographic seizures were defined as rhythmic spikes or sharp-wave discharges with amplitudes at least twice the average amplitude of baseline, frequency at least 2 Hz and duration at least 5 s. Behavioral seizures were also identified by verifying postictal suppression of the baseline EEG activity, which typically occurs after a seizure but is not accompanied by electrographic artifacts associated with mouse behavior other than seizures. Seizure duration and seizure latency (time to occurrence of first seizure after intracortical injections) were calculated from the electrographic seizure data. Seizure severity was graded using a Racine scale as follows: stage 1 (mouth and facial movements); stage 2 (head nodding); stage 3 (forelimb clonus); stage 4 (forelimb clonus and rearing); and stage 5 (forelimb clonus, rearing and falling). Subtle behavioral seizures without forelimb clonus were assigned a seizure score <3. At the end of the experiment, mice were perfused transcardially with saline to remove blood and with 4% paraformaldehyde to fix the brains for IHC analysis to assess the degradation of ECM.

### Gel electrophoresis and western blot

The levels of Kir4.1 and GLT1 in the acute slices after ChABC treatment were measured by western blot analysis with some modifications as described previously^24,25^. In brief, we prepared acute cortical slices, followed by recovery and ChABC-mediated PNN digestion as described above. Cortical tissue from brain slices was isolated and flash-frozen by briefly dropping tubes containing tissue into liquid nitrogen and storing at −80 °C until further processing. We homogenized the tissue using a rotor-stator homogenizer in a lysis buffer (50 mM Tris-HCl, pH 8.00, 150 mM NaCl, 1% Triton X-100, 0.5% sodium deoxycholate, 0.1% SDS, protease inhibitors (P8340, Sigma) and phosphatase inhibitors (P0044, Sigma); 10 µl lysis buffer per mg of tissue), followed by collecting the supernatant and centrifugation (15,000*g*, 20 min, 4 °C). We used bicinchoninic acid (BCA) protein assay (Pierce BCA Protein Assay kit, 23225, Thermo Fisher Scientific) to determine the total protein concentration in the supernatant. Next, we denatured a 10-µg total protein sample at 50 °C for 10 min followed by performing electrophoresis using polyacrylamide gel (4–15% Tris–glycine extended polyacrylamide gel, 567-1085, Bio-Rad). Proteins were transferred from gel to a PVDF membrane, followed by blocking in Tris-buffered saline-based Odyssey blocking buffer (LI-COR) for 1 h at room temperature. Next, we incubated the membrane with rabbit anti-Kir4.1 (1:2,000 dilution, APC-035, Alomone Labs) or guinea pig anti-GLT1 (1:1,500 dilution, AB1783 Millipore) and mouse-anti-β-actin antibody (1:3,000 dilution, MA1-140, Invitrogen) overnight at 4 °C followed by washing and incubation with secondary antibodies (IRDye 800CW donkey anti-guinea pig/rabbit IgG, 0.2 µg ml^−1^, 925-32212 and IRDye 680RD donkey anti-mouse IgG, 0.2 µg ml^−1^, 925-68073; LI-COR) at 1:20,000 dilution for 2 h at room temperature. Secondary antibodies were washed and membranes were imaged using an Odyssey imaging system (LI-COR). Densitometric analysis of protein levels was performed using Image Studio software (LI-COR).

### Immunohistochemistry

Mice were injected with a mixture of ketamine and xylazine (100 mg kg^−1^ and 10 mg kg^−1^, respectively) and subsequently perfused transcardially with PBS followed by 4% PFA. We dissected out the brains and stored them overnight in 4% PFA at 4 °C followed by storing them in PBS at 4 °C until sectioning was performed. We cut 50-μm-thick floating sections using a 5,100 mz vibratome from Campden Instruments or Pelco EasiSlicer from Ted Pella. The sections were either used for IHC immediately or stored at −20 °C in a custom-made storage medium (10% (*v*/*v*) 0.2 mM phosphate buffer, 30% (*v*/*v*) glycerol, 30% (*v*/*v*) ethylene glycol in deionized water, pH 7.2–7.4) for future uses. To minimize procedure-associated variations, we stained duplicate sections from five to seven mice of each experimental group in a single batch. In brief, sections were retrieved from −20 °C storage, rinsed three times with PBS and permeabilized and blocked by incubating them in blocking buffer (0.5% Triton X-100 and 10% goat serum in PBS) for 2 h at room temperature in a 24-well plate. Sections were incubated for 18–24 h at 4 °C with appropriate primary antibodies or biotinylated WFA (B-1355, Vector Laboratories) in diluted blocking buffer (1:3 of blocking buffer and PBS). Following this, we incubated sections with appropriate secondary antibodies and Alexa Fluor 555-conjugated streptavidin (S32355, Thermo Fisher Scientific, 1:500 dilution) in diluted blocking buffer overnight at 4 °C in dark. Further, the sections were rinsed with PBS and were mounted on glass slides (Fisherfinest 25 × 25 × 1, 12-544-2) covered with cover glass and the edges of the slides were sealed with nail polish. Amyloid plaques in 5xFAD^+^ mice sections were stained by Amylo-Glo (TR-300-AG, Biosensis) following the manufacturer’s instructions. All antibodies used in the study were validated either by vendors and/or many published studies. The primary and secondary antibodies used are given in Supplementary Table 1.

### Confocal imaging and analysis

Representative images and data in Figs. 1a–f, 2a–e and 4b–r and Extended Data Figs. 1 and 3) were acquired using Nikon A1 confocal microscope, and quantification was performed by associated NIS-Elements AR analysis program. Images and data in the remaining figures were acquired using an Olympus FV 3000 confocal microscope and images were analyzed using ImageJ. We utilized several different objective lenses, including ×10 (air), ×20 (air), ×40 (oil), ×60 (oil) or ×100 (oil) with a range of optical zoom based on the experimental requirement. Images were acquired as 12 bits and acquisition settings were minimally adjusted to accommodate a few unsaturated and saturated pixels.

To acquire high-magnification images for PNN hole analysis, we excluded the topmost ~2-µm area from the surface due to the occasional uneven tissue surface and restricted our imaging to the top 3–10-µm depth (~8-µm-thick tissue block). Within the above-defined ~8-µm tissue block, we selected the optical plane containing the largest perimeter of a PNN/PV neuron. We used either a ×40 oil immersion objective lens (Plan Fluor ×40 Oil DIC H N2, 1.3 NA, Scanner zoom 4 or 5, 0.2 µm optical section) or ×100 oil immersion (UPlanApo 100XOHR 1.5 NA, 3 or 4 zoom, 0.2-µm optical section, 0.031 µm per pixel) objective lens to take 1,024 × 1,024-sized images. With a range of 405–647 nm light wavelength, the resolution limit of the above objective lenses is 250–310 nm. With the above settings, the lateral and axial resolution exceeded the Nyquist to reliably digitize the optical signal. We adjusted the acquisition settings (laser power, PMT gain and offset) to accommodate the full range of signal (reflected by a few under-saturated and few saturated pixels in the image). Once set, we minimally adjusted the acquisition settings.

#### PNN disruption analysis

The spread of PNN disruption by ChABC injection in mice brains was quantified from whole coronal section images (Fig. 4b,c). We drew uniform-sized regions of interest (ROIs) (0.4 × 0.4 mm^2^) adjacent to each other starting from the ChABC incision site toward the lateral side of the coronal plane. The mean fluorescence intensity was computed. All analyzed images/ROIs at similar distances from the incision site were tabulated to plot the mean and s.d. of the fluorescence intensity. To assess the PNN disruption on PV cells after AAV-mediated Acan KO, 5xFAD model of Alzheimer’s disease and TMEV model of seizure, we selected a 0.8-µm perimeter area of cell soma and binarized the WFA signal using an automated thresholding method (OTSU) in ImageJ and computed WFA intensity and pericellular WFA area (Fig. 5).

#### Analysis of PNN holes

We assessed the PNN holes for the presence of astrocytic processes (Fig. 1) and synaptic contacts (Fig. 2) and their fate after ChABC treatment (Extended Data Fig. 5) using the PNN line intensity profile method with slight modifications as described previously^14,15^. In brief, we acquired high-magnification (×200 or higher) images of individual PNNs at their maximum perimeter plane (Supplementary Fig. 1). Subsequently, we drew a polyline on PNN (WFA) along the entire periphery of the cell and generated an intensity profile consisting of high-intensity peaks (Supplementary Fig. 1b, magenta plot) and low-intensity drops. We set a threshold of WFA intensity (ranging from 40–66% of the unsaturated peak WFA intensity) that covered the maximum number of drops as PNN holes (Supplementary Fig. 1b). We observed that the size of a hole in the line profile may range from several microns to as small as 0.5 µm (Supplementary Fig. 1c, gray bars) depending on whether the image was captured at the center or the edge of a hole. To overcome this subjectivity, we set a cutoff of 0.5 µm (based on the 250–310 nm resolution limit of objective lenses) as the minimum width of a hole to consider for analysis. We moved a 0.5-µm bar through the entire line profile and marked the holes with arrows (Supplementary Fig. 1c, black arrows) for counting purposes. Thus, a minimum 0.5-µm wide WFA intensity drops under the threshold line (Supplementary Fig. 1c–e) were considered as PNN holes.

The presence of a specific fluorophore peak in the PNN hole was determined by the presence of a clearly distinguishable peak within the two consecutive peaks of WFA (Supplementary Fig. 1c–e, gray bars). Subsequently, we provided unique identifying marks (Supplementary Fig. 1c, black downward arrows) to each PNN hole and computed the presence or absence of astrocytic/synaptic components within it. To quantify the degree of perforations in PNNs after ChABC disruption, we counted the number of PNN holes as described above and normalized it to the cell perimeter.

#### PNN disruption and Kir4.1 and GLT1 expressions analysis in fixed acute brain slices

To assess whether ChABC-mediated PNN digestion in acute brain slices alters the expressions of Kir4.1 and GLT1 to influence the astrocytic potassium and glutamate uptake, we fixed acute brain slices after electrophysiological recordings and performed immunostaining using specific antibodies and WFA. The ×40 magnification images were acquired using Olympus FV 3000 and analyzed using ImageJ. The signal of the individual channel (Kir4.1/GLT1, WFA and AldheGFP) was binarized using an inbuilt thresholding function OTSU and the resulting total area was tabulated to plot the graphs.

#### Quantification of astrocytic coverage and synaptic puncta

To quantify the pericellular astrocytic coverage of PV/excitatory neurons, we acquired high-magnification images using either Nikon A1 (40 × 5 optical zoom oil immersion lens) or Olympus Fluoview FV 3000 (100 × 3 or 4, oil immersion objective lens) with a 0.2-µm optical plane thickness. After image acquisition, we generated a binary representation of the cell soma using inbuilt functions in ImageJ and Nikon elements programs. We defined a 0.8-µm wide perimeter from the cell surface as a pericellular area (based on the pericellular width covered by PNN). Subsequently, we binarized the individual channels (AldheGFP, S100B, GLT1 and Kir4.1) using inbuilt auto thresholding functions in Nikon elements or ImageJ (OTSU). Using Boolean operations, we computed the binary areas of different astrocytic markers confined to the cell perimeter defined above (Supplementary Fig. 2). We normalized the pericellular area with the perimeter of the same cell before pooling images from mice.

We added one more step of find maxima in ImageJ or an analogous function in Nikon AR analysis programs to quantify the overall and pericellular numerical densities of vGlut1 and vGAT puncta in the above protocol. A prominence setting of 500 (for vGlut1 puncta) or 2,000 (for vGAT puncta) was found appropriate to capture all puncta and was used for images. The total number of synapses in the entire image was used to plot the total vGlut1/vGAT puncta. We used Boolean operations to compute the total pericellular synapses and pericellular synapses with astrocytic processes in contact with them. The resultant absolute values were normalized to the perimeter of the individual cells and were used for data pooling or directly for plotting graphs.

#### Volumetric analysis in Imaris

The representative 3D rendering videos and 3D reconstruction images of the PNN and pericellular astrocytic processes in Figs. 1, 2, 3 and 4 were generated using Imaris v.9.90 (Oxford Instruments). In brief, we generated volumetric masks from the PV channel that were expanded by 0.8 –1.0 µm to accommodate pericellular PNN structures. These masks were then used to create new astrocytic and synaptic data channels that excluded structures outside of the pericellular domain. The enlarged PV channel volume was created using the surface creation tool with smoothing detail enabled and a surface grain size set to 0.103 µm. Background subtraction was also enabled with the diameter of the largest sphere set to 0.388 µm and manual thresholding set to a value of 200. Astrocytic and synaptic channel volumes were created using the surface creation tool with smoothing detail enabled and a surface grain size set to 0.103 µm. Background subtraction was also enabled with the diameter of the largest sphere set to 0.388 µm and manual thresholding set to 200.

Illustrations and cartoons were created with BioRender.com.

### Statistics and reproducibility

Data in the bar diagrams are expressed as mean ± s.d. unless stated otherwise in the figure. Individual data points are represented by dots. Figure legends contain the essential details, including numerical values of mean, s.d., biological or technical replicates, statistical tests and corrections. The detailed statistical analysis data, including test statistics, *P* values, post hoc comparisons and corrections are summarized in Supplementary Table 2. The sample size was not predetermined but was based on published relevant studies. We ran appropriate normality tests and found that data distribution was sufficiently normal and variance within groups was sufficiently similar to be used for parametric tests. Therefore, experimental designs with two treatment groups were analyzed by two-tailed unpaired *t*-test unless stated otherwise in the figure legends. Welch’s correction was applied regardless of statistically different variances unless stated otherwise. Experimental designs with more than two groups were analyzed using one-way or two-way ANOVA followed by Tukey’s post hoc multiple comparison tests. Statistically significant differences between groups are shown in graphs as *P < 0.05, **P < 0.01, ***P < 0.001 and ****P < 0.0001. No data were excluded. Data analysis was performed using Microsoft Excel and Origin 2021b (OriginLab). For representative experiments, including Figs. 3b,c,j and 6a and Extended Data Figs. 1a,d,f,h, 2a, 5a,b and 9a, we conducted a minimum of three observations in three different mice. Data collection and analysis were performed blind to the conditions of the experiments for data in Figs. 3l–q, 5, 6q and 7l,m and Extended Data Figs. 4 and 6; for remaining data, explicit visual differences in experimental groups prevented us from performing blinded experimentation and analysis.

### Reporting summary

Further information on research design is available in the Nature Portfolio Reporting Summary linked to this article.

## Online content

Any methods, additional references, Nature Portfolio reporting summaries, source data, extended data, supplementary information, acknowledgements, peer review information; details of author contributions and competing interests; and statements of data and code availability are available at 10.1038/s41593-024-01714-3.

### Supplementary information

Supplementary Information Supplementary Fig. 1 (PNN method), 2 (area analysis) and 3 (unprocessed western blot images).

Reporting Summary

Supplementary Table 1 Antibody list and details.

Supplementary Table 2 Details of statistical tests.

Supplementary Video 3 PNN-astrocyte-synapses video.

Supplementary Video 4 PNN-astrocyte-excitatory-inhibitory synapses.

Supplementary Video 5 Control iGluSNFr.

Supplementary Video 6 ChABC iGluSNFr.

Supplementary Video 7 Stage3 seizure on ChABC.

### Source data

Source Data Fig. 1 Source data for Fig. 1.

Source Data Fig. 2 Source data for Fig. 2.

Source Data Fig. 3 Source data for Fig. 3.

Source Data Fig. 4 Source data for Fig. 4.

Source Data Fig. 5 Source data for Fig. 5.

Source Data Fig. 6 Source data for Fig. 6.

Source Data Fig. 7 Source data for Fig. 7.

Source Data Extended Data Figs. 1 and 2–9 Source data for Extended Data Figs. 1 and 2–9.

## Data Availability

Source data are provided with this paper.

## References

[1] Watanabe, K. et al. Three-dimensional organization of the perivascular glial limiting membrane and its relationship with the vasculature: a scanning electron microscope study. *Okajimas Folia Anat. Jpn.* **87**, 109–121 (2010). 21174940 10.2535/ofaj.87.109

[2] Araque, A. et al. Tripartite synapses: glia, the unacknowledged partner. *Trends Neurosci.* **22**, 208–215 (1999). 10322493 10.1016/s0166-2236(98)01349-6

[3] Asztely, F., Erdemli, G. & Kullmann, D. M. Extrasynaptic glutamate spillover in the hippocampus: dependence on temperature and the role of active glutamate uptake. *Neuron* **18**, 281–293 (1997). 9052798 10.1016/s0896-6273(00)80268-8

[4] Parsons, M. P. & Raymond, L. A. Extrasynaptic NMDA receptor involvement in central nervous system disorders. *Neuron* **82**, 279–293 (2014). 24742457 10.1016/j.neuron.2014.03.030

[5] Chaunsali, L., Tewari, B. P. & Sontheimer, H. Perineuronal net dynamics in the pathophysiology of epilepsy. *Epilepsy Curr.* **21**, 273–281 (2021). 34690566 10.1177/15357597211018688 PMC8512927

[6] Tewari, B. P. et al. A glial perspective on the extracellular matrix and perineuronal net remodeling in the central nervous system. *Front. Cell. Neurosci.* **16**, 1022754 (2022). 36339816 10.3389/fncel.2022.1022754 PMC9630365

[7] Fawcett, J. W., Oohashi, T. & Pizzorusso, T. The roles of perineuronal nets and the perinodal extracellular matrix in neuronal function. *Nat. Rev. Neurosci.* **20**, 451–465 (2019). 31263252 10.1038/s41583-019-0196-3

[8] Patel, D. C. et al. Neuron–glia interactions in the pathophysiology of epilepsy. *Nat. Rev. Neurosci.* **20**, 282–297 (2019). 30792501 10.1038/s41583-019-0126-4 PMC8558781

[9] Morawski, M. et al. Ion exchanger in the brain: quantitative analysis of perineuronally fixed anionic binding sites suggests diffusion barriers with ion sorting properties. *Sci. Rep.* **5**, 16471 (2015). 26621052 10.1038/srep16471 PMC4664884

[10] Syková, E. & Nicholson, C. Diffusion in brain extracellular space. *Physiol. Rev.* **88**, 1277–1340 (2008). 18923183 10.1152/physrev.00027.2007 PMC2785730

[11] Pizzorusso, T. et al. Reactivation of ocular dominance plasticity in the adult visual cortex. *Science* **298**, 1248–1251 (2002). 12424383 10.1126/science.1072699

[12] Torres-Ceja, B. & Olsen, M. L. A closer look at astrocyte morphology: Development, heterogeneity, and plasticity at astrocyte leaflets. *Curr. Opin. Neurobiol.* **74**, 102550 (2022). 35544965 10.1016/j.conb.2022.102550 PMC9376008

[13] Ueno, H. et al. Layer-specific expression of extracellular matrix molecules in the mouse somatosensory and piriform cortices. *IBRO Rep.* **6**, 1–17 (2019). 30582064 10.1016/j.ibror.2018.11.006 PMC6293036

[14] Tewari, B. P. & Sontheimer, H. Protocol to quantitatively assess the structural integrity of perineuronal nets ex vivo. *Bio-Protoc.* **9**, e3234 (2019). 33654764 10.21769/BioProtoc.3234 PMC7854210

[15] Tewari, B. P. et al. Perineuronal nets decrease membrane capacitance of peritumoral fast spiking interneurons in a model of epilepsy. *Nat. Commun.* **9**, 4724 (2018). 30413686 10.1038/s41467-018-07113-0 PMC6226462

[16] Freeman, M. R. Specification and morphogenesis of astrocytes. *Science* **330**, 774–778 (2010). 21051628 10.1126/science.1190928 PMC5201129

[17] Brückner, G. et al. Postnatal development of perineuronal nets in wild-type mice and in a mutant deficient in tenascin-R. *J. Comp. Neurol.* **428**, 616–629 (2000). 11077416 10.1002/1096-9861(20001225)428:4<616::aid-cne3>3.0.co;2-k

[18] Patel, D. C. et al. Increased expression of chondroitin sulfate proteoglycans in dentate gyrus and amygdala causes postinfectious seizures. *Brain* **147**, 1856–1870 (2024). 38146224 10.1093/brain/awad430 PMC11068111

[19] Harkness, J. H. et al. Diurnal changes in perineuronal nets and parvalbumin neurons in the rat medial prefrontal cortex. *Brain Struct. Funct.* **226**, 1135–1153 (2021). 33585984 10.1007/s00429-021-02229-4 PMC8086998

[20] Rowlands, D. et al. Aggrecan directs extracellular matrix-mediated neuronal plasticity. *J. Neurosci.* **38**, 10102–10113 (2018). 30282728 10.1523/JNEUROSCI.1122-18.2018 PMC6596198

[21] Somaiya, R. D. et al. Development of astrocyte morphology and function in mouse visual thalamus. *J. Comp. Neurol.* **530**, 945–962 (2022). 34636034 10.1002/cne.25261 PMC8957486

[22] Bergles, D. E. & Jahr, C. E. Synaptic activation of glutamate transporters in hippocampal astrocytes. *Neuron* **19**, 1297–1308 (1997). 9427252 10.1016/s0896-6273(00)80420-1

[23] Hanson, E., Danbolt, N. C. & Dulla, C. G. Astrocyte membrane properties are altered in a rat model of developmental cortical malformation but single-cell astrocytic glutamate uptake is robust. *Neurobiol. Dis.* **89**, 157–168 (2016). 26875663 10.1016/j.nbd.2016.02.012 PMC4794447

[24] Robel, S. et al. Reactive astrogliosis causes the development of spontaneous seizures. *J. Neurosci.* **35**, 3330–3345 (2015). 25716834 10.1523/JNEUROSCI.1574-14.2015 PMC4339349

[25] Kahanovitch, U. et al. MeCP2 deficiency leads to loss of glial Kir4.1. *eNeuro*10.1523/ENEURO.0194-17.2018 (2018). 10.1523/ENEURO.0194-17.2018 PMC5818552 29464197

[26] Campbell, S. C. et al. Potassium and glutamate transport is impaired in scar-forming tumor-associated astrocytes. *Neurochem. Int.* **133**, 104628 (2020). 31825815 10.1016/j.neuint.2019.104628 PMC6957761

[27] Marvin, J. S. et al. An optimized fluorescent probe for visualizing glutamate neurotransmission. *Nat. Methods* **10**, 162–170 (2013). 23314171 10.1038/nmeth.2333 PMC4469972

[28] Rankin‐Gee, E. K. et al. Perineuronal net degradation in epilepsy. *Epilepsia* **56**, 1124–1133 (2015). 26032766 10.1111/epi.13026

[29] Vöglein, J. et al. Seizures in Alzheimer’s disease are highly recurrent and associated with a poor disease course. *J. Neurol.* **267**, 2941–2948 (2020). 32488295 10.1007/s00415-020-09937-7 PMC7501095

[30] Lupori, L. et al. A comprehensive atlas of perineuronal net distribution and colocalization with parvalbumin in the adult mouse brain. *Cell Rep.* **42**, 112788 (2023). 37436896 10.1016/j.celrep.2023.112788

[31] Lawal, O., Ulloa Severino, F. P. & Eroglu, C. The role of astrocyte structural plasticity in regulating neural circuit function and behavior. *Glia* **70**, 1467–1483 (2022). 35535566 10.1002/glia.24191 PMC9233050

[32] Konopka, A. et al. Cleavage of hyaluronan and CD44 adhesion molecule regulate astrocyte morphology via Rac1 signalling. *PLoS ONE* **11**, e0155053 (2016). 27163367 10.1371/journal.pone.0155053 PMC4862642

[33] Kruk, P. K. et al. Astrocytic CD44 deficiency reduces the severity of kainate-induced epilepsy. *Cells* **12**, 1483 (2023). 37296604 10.3390/cells12111483 PMC10252631

[34] Oliferenko, S. et al. Hyaluronic acid (HA) binding to CD44 activates Rac1 and induces lamellipodia outgrowth. *J. Cell Biol.* **148**, 1159–1164 (2000). 10725329 10.1083/jcb.148.6.1159 PMC2174315

[35] Bourguignon, L. Y. Hyaluronan-mediated CD44 activation of RhoGTPase signaling and cytoskeleton function promotes tumor progression. *Semin. Cancer Biol.* **18**, 251–259 (2008). 18450475 10.1016/j.semcancer.2008.03.007 PMC2505114

[36] Vedunova, M. et al. Seizure-like activity in hyaluronidase-treated dissociated hippocampal cultures. *Front. Cell. Neurosci.* **7**, 149 (2013). 24062641 10.3389/fncel.2013.00149 PMC3770920

[37] Balashova, A. et al. Enzymatic digestion of hyaluronan-based brain extracellular matrix in vivo can induce seizures in neonatal mice. *Front. Neurosci.* **13**, 1033 (2019). 31632233 10.3389/fnins.2019.01033 PMC6779145

[38] Young, I. J. Reversible seizures produced by neuronal hyaluronic acid depletion. *Exp. Neurol.* **8**, 195–202 (1963). 14272260

[39] Arranz, A. M. et al. Hyaluronan deficiency due to Has3 knock-out causes altered neuronal activity and seizures via reduction in brain extracellular space. *J. Neurosci.* **34**, 6164–6176 (2014). 24790187 10.1523/JNEUROSCI.3458-13.2014 PMC4004806

[40] Dityatev, A. & Fellin, T. Extracellular matrix in plasticity and epileptogenesis. *Neuron Glia Biol.* **4**, 235–247 (2008). 19497143 10.1017/S1740925X09000118

[41] Saghatelyan, A. K. et al. Reduced perisomatic inhibition, increased excitatory transmission, and impaired long-term potentiation in mice deficient for the extracellular matrix glycoprotein tenascin-R. *Mol. Cell. Neurosci.* **17**, 226–240 (2001). 11161481 10.1006/mcne.2000.0922

[42] Comper, W. D. & Laurent, T. C. Physiological function of connective tissue polysaccharides. *Physiol. Rev.* **58**, 255–315 (1978). 414242 10.1152/physrev.1978.58.1.255

[43] Hrabětová, S. et al. Calcium diffusion enhanced after cleavage of negatively charged components of brain extracellular matrix by chondroitinase ABC. *J. Physiol.* **587**, 4029–4049 (2009). 19546165 10.1113/jphysiol.2009.170092 PMC2756436

[44] Manning, G. S. *Polyelectrolytes*. *Annu. Rev. Phys. Chem.* **23**, 117–140 (1972).

[45] Dubey, D. et al. Increased metalloproteinase activity in the hippocampus following status epilepticus. *Epilepsy Res.* **132**, 50–58 (2017). 28292736 10.1016/j.eplepsyres.2017.02.021 PMC6690398

[46] McRae, P. A. & Porter, B. E. The perineuronal net component of the extracellular matrix in plasticity and epilepsy. *Neurochem. Int.* **61**, 963–972 (2012). 22954428 10.1016/j.neuint.2012.08.007 PMC3930202

[47] Gonzalez-Burgos, G., Fish, K. N. & Lewis, D. A. GABA neuron alterations, cortical circuit dysfunction and cognitive deficits in Schizophrenia. *Neural Plast.* **2011**, 723184 (2011). 21904685 10.1155/2011/723184 PMC3167184

[48] Brückner, G., Morawski, M. & Arendt, T. Aggrecan-based extracellular matrix is an integral part of the human basal ganglia circuit. *Neuroscience* **151**, 489–504 (2008). 18055126 10.1016/j.neuroscience.2007.10.033

[49] Gittis, ArynH. et al. Rapid target-specific remodeling of fast-spiking inhibitory circuits after loss of dopamine. *Neuron* **71**, 858–868 (2011). 21903079 10.1016/j.neuron.2011.06.035 PMC3170520

[50] Dityatev, A., Seidenbecher, C. I. & Schachner, M. Compartmentalization from the outside: the extracellular matrix and functional microdomains in the brain. *Trends Neurosci.* **33**, 503–512 (2010). 20832873 10.1016/j.tins.2010.08.003

