# Supplementary material for: Astrocytes require perineuronal nets to maintain synaptic homeostasis in mice
Source: Nat Neurosci. 2024 Jul 17;27(8):1475–88. doi: 10.1038/s41593-024-01714-3 (PMC11303255; doi:10.1038/s41593-024-01714-3)
Supplement: Supplementary file 1 — Supplementary Fig. 1 (PNN method), 2 (area analysis) and 3 (unprocessed western blot images). [file 41593_2024_1714_MOESM1_ESM.pdf]

nature neuroscience

Supplementary information https://doi.org/10.1038/s41593-024-01714-3

Astrocytes require perineuronal nets to maintain synaptic homeostasis in mice

In the format provided by the authors and unedited

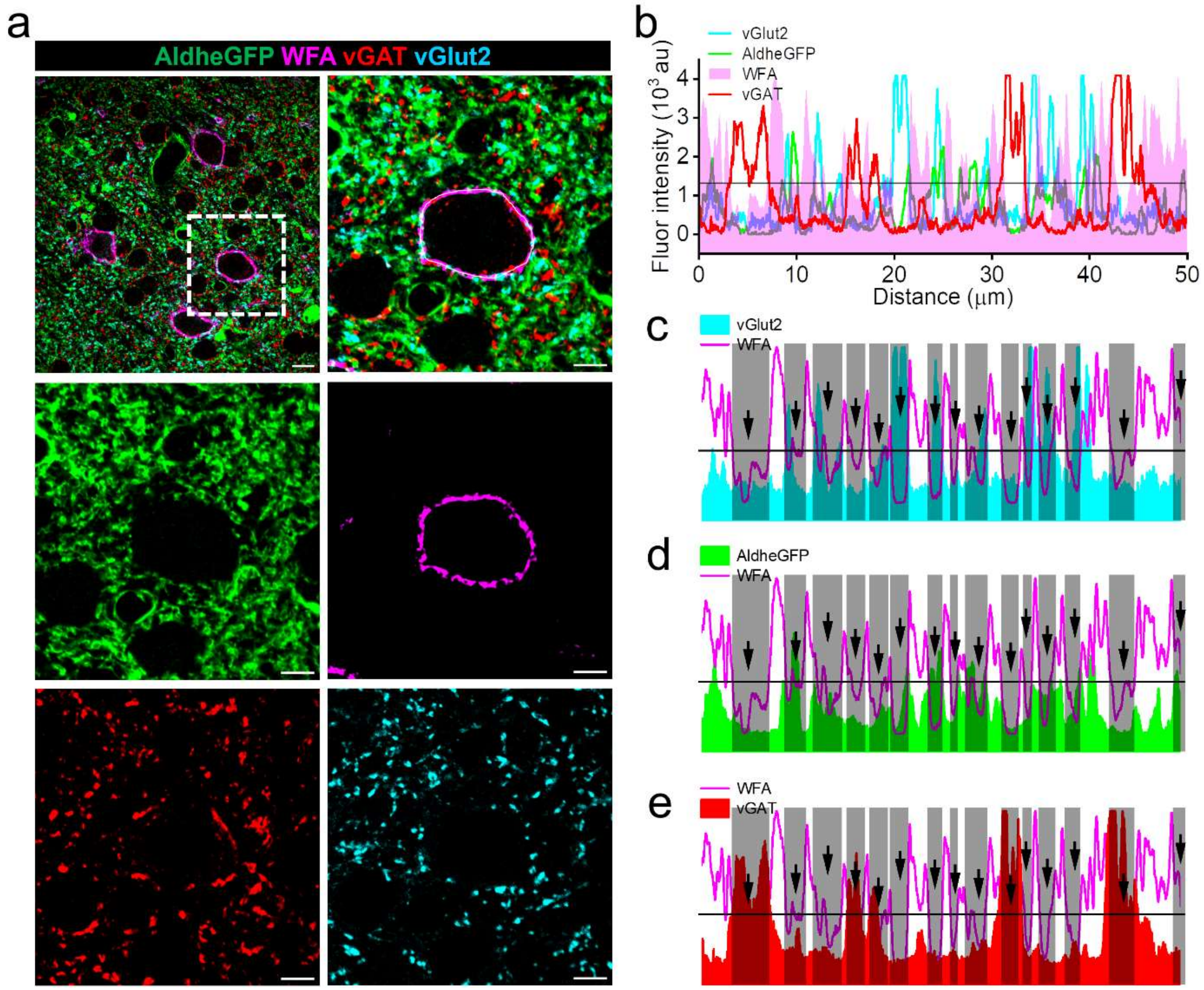

Supplementary Fig. 1 Method of PNN holes analysis to determine astrocytic and synaptic contacts.

a Confocal micrograph (top right) showing immunofluorescence of astrocyte (AldheGFP - green), PNN (WFA - magenta), GABAergic (vGAT - red) and glutamatergic (vGlut2 - cyan) presynaptic terminals in mouse cerebral cortex. Magnified images of a single PNN (marked white rectangle) and various markers are shown in the bottom and right panel images. Scale 10µm top right image, 5µm in magnified images.

b Fluorescence intensity profile of a polyline drawn on the PNN (WFA) (top right image in **a**) showing distinct peaks and troughs patterns. A black line on the line profiles indicates the threshold intensity of WFA. Troughs in the WFA line profile that are below threshold are considered PNN holes. The WFA area under the line intensity profile is filled for visualization purposes.

c - e Line intensity profiles of WFA with vGlut2 (**c**), AldheGFP (**d**), and vGAT (**e**). PNN holes are indicated by arrows. Gray bars indicate the area of the PNN hole in which peaks of vGlut2 (**c**), AldheGFP (**d**), and vGAT (**e**) are intersecting to suggest their expression in the PNN holes.

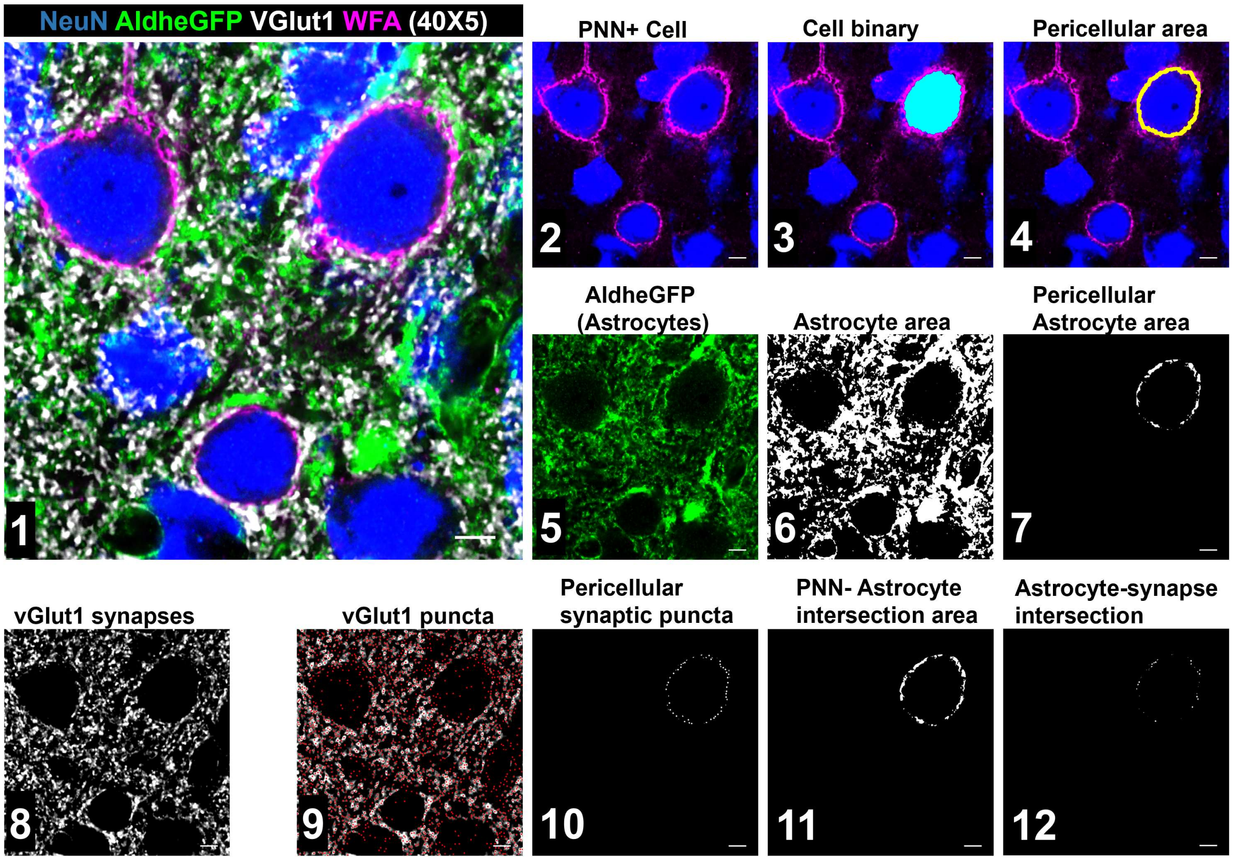

Supplementary Fig. 2 Pericellular astrocytic coverage and synaptic contacts analysis method.

Multichannel confocal image (1), showing immunofluorescence labeling of neurons (NeuN - blue), astrocytes (AldheGFP - green), PNNs (WFA - magenta), and synaptic terminals (vGlut1 - gray). The NeuN signal of the soma of PNN-expressing neurons (2), is binarized (3), and the pericellular 0.8 - 1µm area (4) is defined. AldheGFP signal (5), is binarized using automated OTSU function (6), and pericellular AldheGFP area (7), is extracted by intersecting (4) and (6) binary images. Synaptic marker vGlut1 (8), image is processed with an automated peak detection function to detect vGlut1 puncta (8). Intersecting (9) with (4) generates pericellular synaptic puncta (10). Intersecting the PNN signal with (7) generates a PNN-astrocyte intersection area (11), and intersecting (7) with (10) generates vGlut1 puncta in contact with the pericellular astrocytic area (12). Scale bar: 5µm.

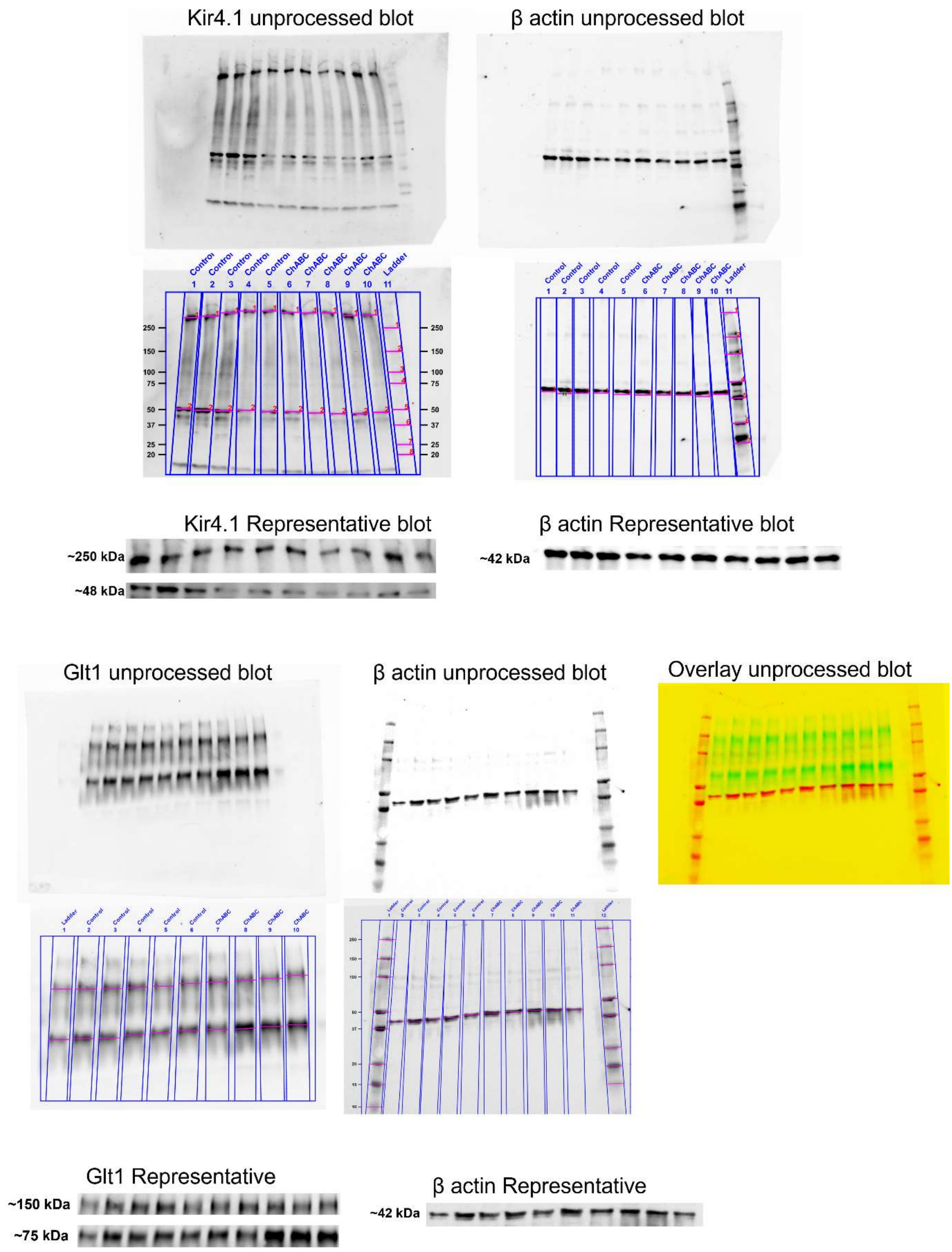

Supplementary Fig. 3 Unprocessed western blot images.
