# Supplementary material for: Astrocytes require perineuronal nets to maintain synaptic homeostasis in mice
Source: Nat Neurosci. 2024 Jul 17;27(8):1475–88. doi: 10.1038/s41593-024-01714-3 (PMC11303255; doi:10.1038/s41593-024-01714-3)
Supplement: Supplementary file 2 — Reporting Summary [file 41593_2024_1714_MOESM2_ESM.pdf]

Corresponding author(s): Harald Sontheimer
Last updated by author(s): Jun 12, 2024

Reporting Summary

Nature Portfolio wishes to improve the reproducibility of the work that we publish. This form provides structure for consistency and transparency in reporting. For further information on Nature Portfolio policies, see our Editorial Policies and the Editorial Policy Checklist.

Statistics

For all statistical analyses, confirm that the following items are present in the figure legend, table legend, main text, or Methods section.

n/a	Confirmed	
☐	☒	The exact sample size (*n*) for each experimental group/condition, given as a discrete number and unit of measurement
☒	☐	A statement on whether measurements were taken from distinct samples or whether the same sample was measured repeatedly
☐	☒	The statistical test(s) used AND whether they are one- or two-sided<br>*Only common tests should be described solely by name; describe more complex techniques in the Methods section.*
☐	☒	A description of all covariates tested
☐	☒	A description of any assumptions or corrections, such as tests of normality and adjustment for multiple comparisons
☐	☒	A full description of the statistical parameters including central tendency (e.g. means) or other basic estimates (e.g. regression coefficient) AND variation (e.g. standard deviation) or associated estimates of uncertainty (e.g. confidence intervals)
☐	☒	For null hypothesis testing, the test statistic (e.g. *F*, *t*, *r*) with confidence intervals, effect sizes, degrees of freedom and *P* value noted<br>*Give P values as exact values whenever suitable.*
☒	☐	For Bayesian analysis, information on the choice of priors and Markov chain Monte Carlo settings
☒	☐	For hierarchical and complex designs, identification of the appropriate level for tests and full reporting of outcomes
☐	☒	Estimates of effect sizes (e.g. Cohen's *d*, Pearson's *r*), indicating how they were calculated

Our web collection on statistics for biologists contains articles on many of the points above.

Software and code

Policy information about availability of computer code

Data collection	we used Clampex 10.4 (Molecular Devices), Olympus Fluoview FV3000, NIS-Elements AR 5.11, AcqKnowledge 5.0 (BIOPAC Systems), Media Recorder 4.0 (Noldus Information Technology), and Odyssey imaging system (LI-COR) software for data collection. No special codes were used.
Data analysis	We used Microsoft office, Clampfit 10.6 and 11.2 (Molecular Devices), Origin 2021b, Olympus Fluoview FV3000, NIS-Elements AR 5.11, ImageJ, Imaris v9.90 (Oxford Instruments), Observer XT 14.1 (Noldus Information Technology), and Image lab 4.1 (Bio Rad) for data analysis. No special codes were used.

For manuscripts utilizing custom algorithms or software that are central to the research but not yet described in published literature, software must be made available to editors and reviewers. We strongly encourage code deposition in a community repository (e.g. GitHub). See the Nature Portfolio guidelines for submitting code & software for further information.

Data

Policy information about availability of data

All manuscripts must include a data availability statement. This statement should provide the following information, where applicable:

- Accession codes, unique identifiers, or web links for publicly available datasets
- A description of any restrictions on data availability
- For clinical datasets or third party data, please ensure that the statement adheres to our policy

We have provided source data for all main and extended data figures. A data availability statement is provided as "The source data of all main and extended data figures is provided in the manuscript.".

Research involving human participants, their data, or biological material

Policy information about studies with human participants or human data. See also policy information about sex, gender (identity/presentation), and sexual orientation and race, ethnicity and racism.

Reporting on sex and gender	N/A
Reporting on race, ethnicity, or other socially relevant groupings	N/A
Population characteristics	N/A
Recruitment	N/A
Ethics oversight	N/A

Note that full information on the approval of the study protocol must also be provided in the manuscript.

Field-specific reporting

Please select the one below that is the best fit for your research. If you are not sure, read the appropriate sections before making your selection.

☒ Life sciences ☐ Behavioural & social sciences ☐ Ecological, evolutionary & environmental sciences

For a reference copy of the document with all sections, see nature.com/documents/nr-reporting-summary-flat.pdf

Life sciences study design

All studies must disclose on these points even when the disclosure is negative.

Sample size	Sample size was not predetermined, instead we used similar published studies including PMIDs: 33431847, 28712654, 33585984, 30282728, and 30413686 as references for the appropriate sample size.
Data exclusions	No data was excluded.
Replication	In several independent experiments (Figs. 1h, 1l, 1p, 1t, 2d, 2h, 2l, 2p, and 2t) we observed that the means of one or more common experimental group/s (such as AldheGFP, Occupied holes etc.) were similar and within ~±10% range. These experiments were not intended to replicate each other; we simply reported our observations.
Randomization	We randomly assigned a cohort of similar aged male and female mice with different experimental groups.
Blinding	In several experiments, explicit visual differences between control and experimental groups did not allow us to do blind experimentation and analysis. However, we did blind experimentation and analysis on the rest of the data (Figs.3l-q, 5, 6q, 7l-m, Ext. data Fig. 4, and Ext. data Fig. 6) in which no such visual differences were observed.

Reporting for specific materials, systems and methods

We require information from authors about some types of materials, experimental systems and methods used in many studies. Here, indicate whether each material, system or method listed is relevant to your study. If you are not sure if a list item applies to your research, read the appropriate section before selecting a response.

Materials & experimental systems

n/a	Involved in the study
☐	☒ Antibodies
☒	☐ Eukaryotic cell lines
☒	☐ Palaeontology and archaeology
☐	☒ Animals and other organisms
☒	☐ Clinical data
☒	☐ Dual use research of concern
☒	☐ Plants

Methods

n/a	Involved in the study
☒	☐ ChIP-seq
☒	☐ Flow cytometry
☒	☐ MRI-based neuroimaging

Antibodies

Antibodies used: All antibodies utilized in the study are enlisted in the supplementary table 1. The details including, vendor, catalogue number, concentration, etc., are present in the same table.

Validation: All primary antibodies are validated by the respective vendors or other investigators in previously published studies. Validation method / evidence has been updated in supplementary table 1.

Animals and other research organisms

Policy information about studies involving animals; ARRIVE guidelines recommended for reporting animal research, and Sex and Gender in Research

Laboratory animals: All mice used in the study were approved by Institutional Animal Care & Use Committee of University of Virginia. We used adult and early postnatal male and female Aldhe1L1eGFP FVB/N (GENSAT project), Pvalb-tdTomato(C57BL/6-Tg 15Gfng/J, Strain #:027395-JAX), Acan fl/fl (CC57BL/6N-Acantm1c(EUCOMM)Hmgu/H mice (strain ID # EM:10224)), Nestin-Cre ((B6.Cg-Tg(Nes-cre)1Kln/J, (Strain #:003771-JAX)), 5XFAD (B6SJL-Tg (APPSwFlLon,PSEN1*M146L*L286V) 6799Vas/Mmjax, Strain # 034840-JAX)), C57BL/6J (strain # 000664), and FVB/NJ mice (strain # 001800).

Wild animals: No wild animals were used in the study.

Reporting on sex: Both sexes were used.

Field-collected samples: Not applicable

Ethics oversight: Animal care and use committee of the University of Virginia

Note that full information on the approval of the study protocol must also be provided in the manuscript.

Plants

Seed stocks: N/A

Novel plant genotypes: N/A

Authentication: N/A
